# Picophytoplankton implicated in productivity and biogeochemistry in the North Pacific Transition Zone

**DOI:** 10.1128/msystems.00801-25

**Published:** 2026-01-16

**Authors:** Rebecca S. Key, Sacha N. Coesel, Mary R. Gradoville, Rhonda L. Morales, Hanna Farnelid, Jonathan P. Zehr, E. Virginia Armbrust, Bryndan P. Durham

**Affiliations:** 1 Department of Biology, Genetics Institute, University of Florida 3463, Gainesville, Florida, USA; 2 School of Oceanography, University of Washington 7284, Seattle, Washington, USA; 3 Columbia River Inter-Tribal Fish Commission 11063, Portland, Oregon, USA; 4 Ocean Sciences Department, University of California Santa Cruz 8787, Santa Cruz, California, USA; 5 Centre for Ecology and Evolution in Microbial Model Systems, Linnaeus University 4180, Kalmar, Sweden; Stellenbosch University, Stellenbosch, South Africa

**Keywords:** marine microbiology, phytoplankton, microbial interactions, biogeochemistry, North Pacific, ocean, mixed models, co-occurrence networks, bioinformatics, net community production

## Abstract

Marine phytoplankton are central to global seascapes, acting as key conduits in element cycling and oceanic food webs. Phytoplankton cell size spans several orders of magnitude (0.2 to >200 µm) and is an important trait that governs metabolism. However, the vast taxonomic diversity within phytoplankton size classes makes it challenging to link specific taxa to bulk community changes in productivity and elemental stoichiometry. To explore phytoplankton biogeography and biogeochemical roles in field populations, we analyzed 3 years of 16S and 18S rRNA gene amplicon sequencing variant (ASV) data alongside biochemical measurements across the dynamic latitudinal gradient of the North Pacific Transition Zone. We identified picophytoplankton community members associated with patterns in net community production (NCP), particulate organic carbon (POC), and particulate organic nitrogen (PON) and uncovered co-occurring species that may influence their growth and abundance. Multivariate linear mixed modeling revealed that the occurrence of chlorophytes explained 22.6% of NCP values, followed by stramenopiles and cyanobacteria. In contrast, POC and PON spatial patterns were best explained by chlorophyte and dinoflagellate spatial patterns. Weighted co-expression network analysis further showed NCP, POC, and PON correlations with a subset of ~40 ASVs belonging to chlorophytes, cyanobacteria, stramenopiles, haptophytes, and dinoflagellates that range in trophic strategy. Association network inference recapitulated these findings and revealed additional co-occurring phytoplankton, grazers, and heterotrophic bacteria. Together, our integrated computational analyses identified key picophytoplankton and co-occurring mixotrophs as major contributors to shaping regional biogeochemical dynamics in the North Pacific Ocean.

**IMPORTANCE:**

Phytoplankton mediate key biogeochemical processes in dynamic oceanic transition zones. However, their vast cell size range and taxonomic diversity make it challenging to link specific taxa to bulk community changes in productivity and elemental stoichiometry. By integrating molecular and biogeochemical measurements from the North Pacific Transition Zone using combined network and multivariate modeling, we identified specific picophytoplankton strongly linked to community production and organic nutrients levels. These picophytoplankton included specific members of cyanobacteria, pelagophytes, haptophytes, and chlorophytes and formed tight associations with several nano- and pico-sized protistan mixotrophs, highlighting how top-down interactions and microbial consortia shape community structure and elemental fluxes. Our work establishes key microbial players that may control fundamental ecosystem processes like carbon and nitrogen cycling and offers a computational framework to track and identify “microbial neighborhoods” that underpin biogeochemical features of an ecosystem.

## INTRODUCTION

Photosynthetic unicellular organisms, known as phytoplankton, are the dominant players in regulating the dynamic interplay of carbon fixation, respiration, and sequestration across marine ecosystems (1). Phytoplankton cell size typically spans 0.2 µm to 1 mm, although some species can form colonies up to 1 cm in diameter. This physical size trait imposes constraints on nutrient uptake and transformation (2, 3). From a single-cell perspective, marine phytoplankton are commonly partitioned into three size classes based on cell diameter: pico- (0.2–2 µm), nano- (2–20 µm), and micro-size (20–200 µm) (4, 5).

In the open ocean, the majority of phytoplankton biomass is attributed to pico-sized phytoplankton, composed of cyanobacteria and the smallest eukaryotes (6). Cyanobacterial picophytoplankton primarily consist of members belonging to the genera *Prochlorococcus* (7) and *Synechococcus* (8). Meanwhile, eukaryotic members in this size range are more taxonomically diverse and consist of members spanning Haptophyta, Chlorophyta, Bacillariophyta, and Dinophyceae groups (9). Picoeukaryotic cells (~1.5  µm diameter) are larger than *Prochlorococcus* (~0.6  µm) and *Synechococcus* (~1  µm), with volumes up to two orders of magnitude greater (10). This size difference, coupled with similar doubling times, helps explain why picoeukaryotes contribute up to an estimated 69% of global picophytoplankton biomass, compared to 17%–39% for *Prochlorococcus* and 12%–15% for *Synechococcus* (11). *Prochlorococcus* numerically dominates oligotrophic gyres, whereas *Synechococcus* and photosynthetic picoeukaryotes become more dominant in mesotrophic, coastal, and high-latitude regions. The high surface-to-volume ratios of cyanobacterial and eukaryotic picophytoplankton enhance nutrient uptake efficiency, providing a competitive advantage within their respective environments (12). Current model simulations estimate their collective contribution of roughly 58% (20% for cyanobacteria and 38% for eukaryotic picophytoplankton) to global net primary production within tropical, subtropical, and mid-latitude regions (13). In contrast, high-latitude regions with elevated nutrients and productivity are typically dominated by micro-sized phytoplankton biomass (14).

While phytoplankton distribution and productivity patterns are characterized across major ocean biomes (15), some regions exhibit greater variability in cell size, taxonomic composition, and nutrient dynamics. For example, oceanic transition zones are boundary regions between ocean biomes where surface convergence creates steep physical and chemical fronts that drive complex dynamics in phytoplankton size structure and taxonomy (16). One such zone, the North Pacific Transition Zone (NPTZ), separates the nitrogen-poor, oligotrophic North Pacific Subtropical Gyre (NPSG) from the nitrogen-replete, iron-deplete Subarctic Gyre (17, 18). *Prochlorococcus* typically dominates the NPSG community (17), while *Synechococcus* and photosynthetic picoeukaryotes become more prevalent within the NPTZ and beyond the subarctic front (18–20). Additionally, O_2_-argon-based estimates of net community production (NCP) in the NPTZ are strongly correlated with increases in nano-phytoplankton, which consist of small diatoms, dinoflagellates, and haptophytes (18). Juranek et al. (18) hypothesized that the observed correlation arose from a combination of increasing nutrients and grazing pressure on picophytoplankton that together facilitated the co-occurrence of pico- and nano-phytoplankton. While these findings link community size structure to elevated NCP, the specific eukaryotic taxa that drive biogeochemical variability across time and space remain poorly resolved.

**Fig 1:**
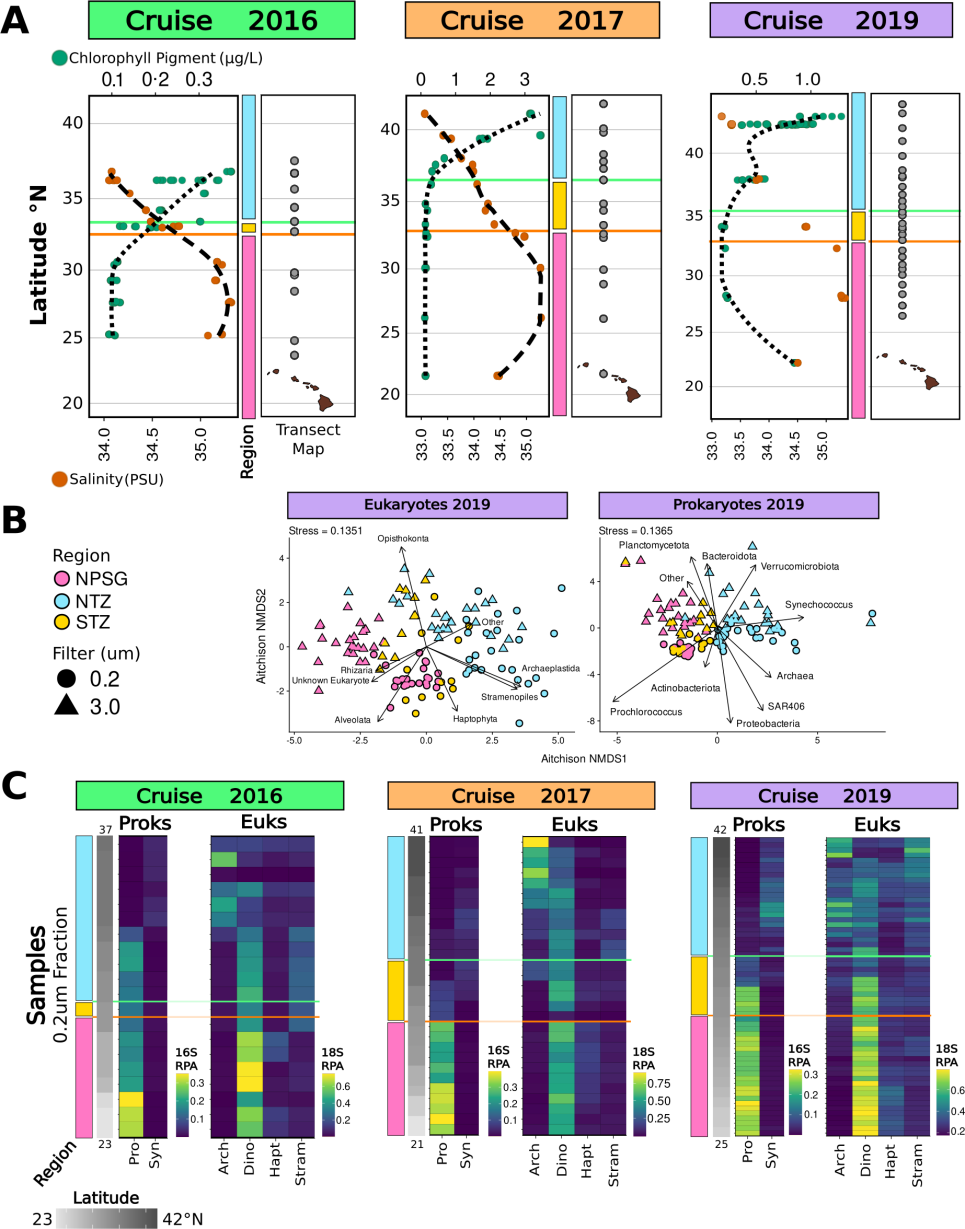
Overview of sampling and microbial distributions across the transect. (**A**) Locations of the 34.82 isohaline salinity front (orange line; 32.15°N–32.5°N across years) and 0.15 mg m^−3^ chlorophyll front (green line; 33°N–36.2°N across years) for 2016, 2017, and 2019 cruises (left), alongside ASV sampling sites (right; dark gray points). Dual X-axis plots display surface salinity (orange points) and chlorophyll (green points) at 15 m depth; dotted and dashed lines show chlorophyll and salinity trends, respectively. Color bars mark regions: North Pacific Subtropical Gyre (NSPG, pink), Southern NPTZ (STZ, yellow), and Northern NPTZ (NTZ, blue), defined by chlorophyll and salinity front positions. (**B**) NMDS of 2019 18S ASV samples collected from 0 to 125 m depths. Circles indicate the small size fraction (0.2–3.0 µm), and triangles indicate the large size fraction (>3.0 µm), colored by region. Stress values are shown top-left. Arrows indicate correlations between samples and the relative percent abundance of taxonomic groups. (**C**) Relative percent abundance (RPA) heatmaps of small size fraction samples collected from 0 to 15 m depths, arranged from the bottom by increasing latitude. Columns represent phytoplankton-containing groups. Prokaryotic (Proks) and eukaryotic (Euks) amplicon data sets are displayed separately, with percent abundance of groups calculated relative to their corresponding community. The left heatmap in each survey set displays relative percent abundances for *Prochlorococcus* (Pro) and *Synechococcus* (Syn) within 16S ASVs, while the right heatmap shows Archaeplastida (Arch), Dinoflagellata (Dino), Haptophyta (Hapt), and Stramenopiles (Stram) within 18S ASVs. A color scale indicating relative percent abundance values for each data set (16S prokaryotes and 18S eukaryotes) is provided at the bottom right. Two sample feature bars to the far left of each heatmap indicate latitude (lower latitudes are light gray, higher latitudes are dark gray, with minimum and maximum latitudes noted) and region information as described in panel **A**, with salinity (orange) and chlorophyll (green) fronts marked by lines.

In this work, we investigate patterns in plankton communities and their relationships with concurrent biogeochemical features from samples collected in 2016, 2017, and 2019 during late spring and early summer along a latitudinal gradient spanning the NPSG and the NPTZ (Fig. 1A). Two frontal features define gradients in the region: (i) a spatially stable salinity front (~34.82 ppt) marking the northern edge of the NPSG, and (ii) a more spatially variable chlorophyll-*a* front (0.15 mg m^−^³) that seasonally migrates from 32°N in winter to 42°N in summer, which signals an increased phytoplankton abundance and separates the southern (STZ) and northern (NTZ) subregions within the NPTZ (18, 21). We used amplicon sequence variant (ASV) data derived from 18S and 16S rRNA gene sequences as proxies to infer relative abundance and community composition of microbial taxa. These data were paired with existing O_2_-argon-based NCP, particulate organic carbon (POC), and particulate organic nitrogen (PON) measurements (18). To investigate relationships between ASVs and biogeochemical components, we applied an integrated network and modeling strategy, which combines multivariate linear mixed modeling (MLMM), weighted gene co-expression network analysis (WGCNA), and sparse inverse covariance estimation for ecological association inference (Spiec-Easi). Collectively, these approaches allowed us to assess plankton ASV contributions to NCP, POC, and PON variability along the transect and identify potential species associations that contribute to population dynamics.

## RESULTS

### Spatial and temporal patterns

We examined spatiotemporal patterns of the small (0.2–3 µm) and large (>3.0 µm) size fraction 18S and 16S ASVs based on broad phylogenetic classifications representative of the most prevalent microbial groups (Fig. 1). Samples spanned depths from 0 to 125 m, with the majority of samples collected from surface waters (15 m) (Fig. S1). Taxonomic groups contributing <1% to total relative abundance were pooled as “Other” (Fig. S2 and S3). Non‐metric multidimensional scaling (NMDS) ordination and analysis of similarities (ANOSIM) revealed the separation of samples by region (R = 0.20–0.50, *P* = 0.001–0.005) and filter (R = 0.19–0.45, *P* = 0.001–0.003), while depth effects were weaker or non‐significant (R = 0.15–0.23) (Fig. 1B; Table S1). NMDS with environmental fitting (EnvFit) revealed the influence of eukaryotic and prokaryotic taxa on ordination placement of samples (Fig. 1B; Table S2). The eukaryotic groups Opisthokonta, Alveolata, and Archaeplastida consistently accounted for a majority of variation in the ordination, with r^2^ values of 84%, 76%, and 73%, respectively (Table S2). Additional contributions came from Haptophyta (r^2^ = 23%–39%) and Stramenopiles (r^2^ = 7%–60%). Of the prokaryotic groups, *Prochlorococcus*, Proteobacteria, Bacteroidota, and *Synechococcus* contributed most to sample ordinates, with r^2^ values reaching 56%, 48%, 46%, and 44%, respectively (Table S2).

Plankton community composition varied markedly between size fractions and regions across the three sampling surveys (Fig. 1B; Tables S1 and S2). Opisthokonta (zooplankton, fungi, and non-zooplankton metazoans) was a major contributor to sample separation in the NMDS ordination (Fig. 1B) and was consistently among the most represented groups in the large size fraction, with an average relative read abundance of 21% across samples from the three cruises (Fig. S2). Alveolata (primarily planktonic dinoflagellates and ciliates, plus parasitic Apicomplexa) were similarly represented in the small and large size fractions, reaching the highest relative abundance in the small size fraction of the NPSG (62% in 2016, 59% in 2017, and 54% in 2019). Archaeplastida (primarily chlorophytes, rhodophytes, and glaucophytes), another strong contributor to eukaryotic sample ordination, was consistently enriched in the NTZ in the small size fraction, with a mean relative abundance of 20% in 2016, 70% in 2017, and 29% in 2019. For prokaryotes, *Prochlorococcus* was the strongest contributor to NMDS sample ordination (Fig. 1B) with consistently high relative abundance in the small size fraction in the NPSG: 30% in 2016, 37% in 2017, and 30% in 2019 (Fig. S3). Proteobacteria dominated each region consistently across years, comprising over 50% of the regional relative abundance within the small size fraction. *Synechococcus* was primarily concentrated in the NTZ, with relatively equal representation across both size fractions, consistent with prior observations of their aggregation and attachment to particles (22, 23), with mean relative abundances of 2% in 2016, 5% in 2017, and 5%–6% in 2019.

To more closely examine community dynamics of phytoplankton across regions, we focused on taxonomies predominantly containing photosynthetic community members (i.e., *Prochlorococcus*, *Synechococcus*, Archaeplastida, Haptophyta, Dinoflagellata within the Alveolata*,* and photosynthesizing Stramenopiles belonging to diatom classes Coscinodiscophyceae, Bacillariophyceae, and Mediophyceae and classes Chrysophyceae, Bolidophyceae, Dictyochophyceae, Pelagophyceae, and Pinguiophyceae). Across most groups, relative abundances of these taxonomies were consistently higher in the small size fraction (Fig. S4), compared to the large size fraction that contained a higher proportion of Opisthokonta reads (Fig. S2). Archaeplastida were 2-fold to 6-fold more enriched in the small fraction, Haptophyta 2-fold to 3-fold, *Prochlorococcus* 4-fold, and Stramenopiles 2-fold to 5-fold. In contrast, Dinoflagellata and *Synechococcus* were relatively evenly distributed across both size fractions. Examining the relative abundances in the small size fraction across the three years revealed recurring spatial patterns across major phytoplankton taxa (Fig. 1C). Indeed, *Prochlorococcus* and Dinoflagellata consistently displayed the highest relative abundance in the NPSG, with Haptophyta contributing to a lesser extent. In contrast, Stramenopiles, Archaeplastida, and *Synechococcus* were more prevalent in the STZ and NTZ.

### Persistent and ephemeral amplicons

**Fig 2:**
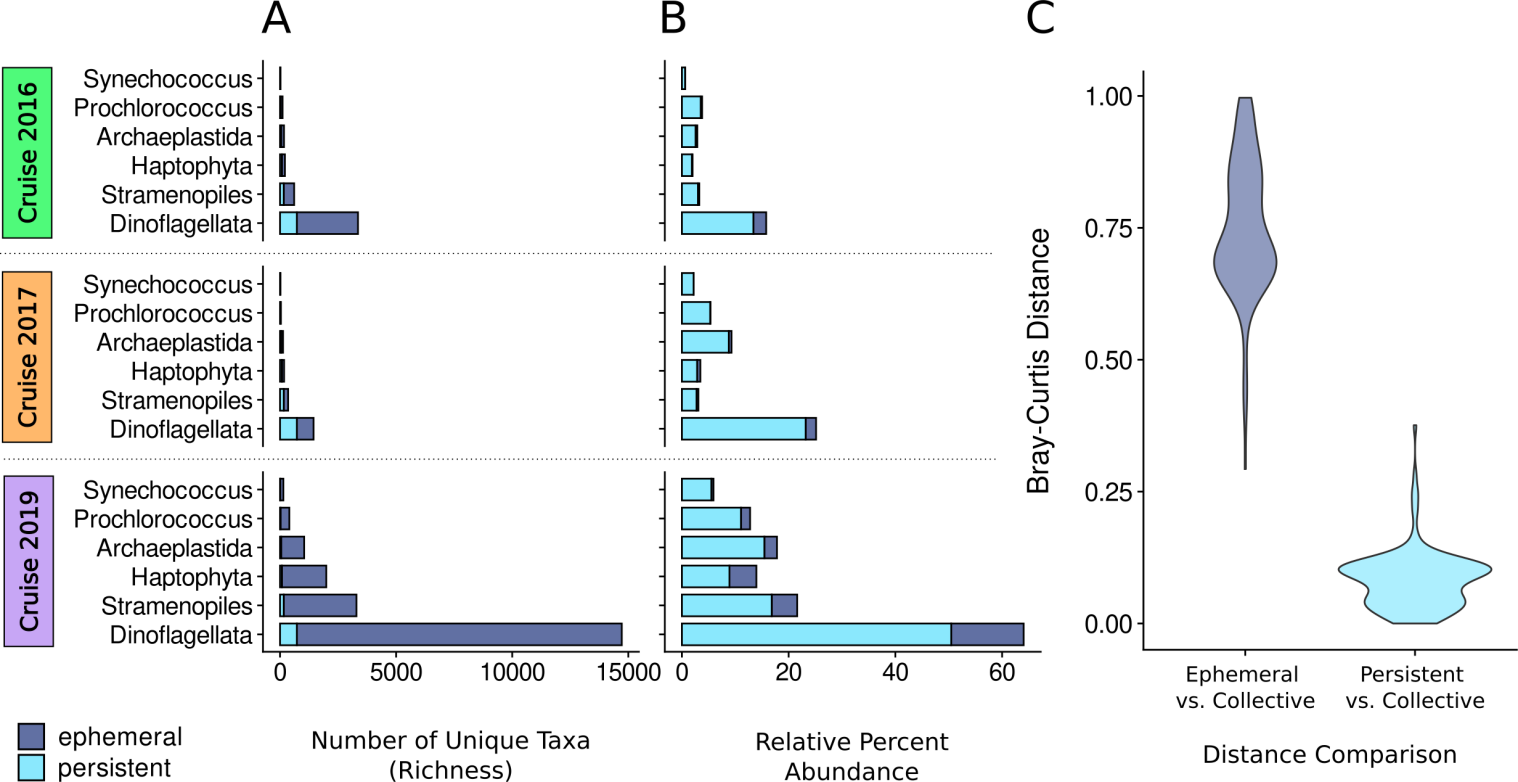
Persistent and ephemeral ASV comparison across yearly surveys. Bar plots show the number of unique ASVs (richness) (**A**) and relative percent abundance of ASVs (**B**) across six phytoplankton-containing groups for 2016, 2017, and 2019 cruises. Richness is divided into persistent (cyan) and ephemeral (dark blue) categories, with persistent ASVs defined as those present in all three cruises and ephemeral ASVs defined as those identified in < 3 cruises. Samples collected from all depths are considered. (**C**) Violin plots show Bray-Curtis distances between ephemeral (left) and persistent (right) group compositions and the overall collective community across all years. Each distribution represents the dissimilarity between ephemeral or persistent groups and the overall collective community, with higher distance values indicating greater dissimilarity from the overall relative percent abundance.

We assessed ASV richness (i.e., the number of unique ASVs) within the six major phytoplankton-containing groups (Fig. 2A) to determine whether the observed spatial patterns across eukaryotic and prokaryotic phytoplankton were driven by “persistent” (i.e., ASVs detected in all years) or “ephemeral” (i.e., ASVs detected in only 1 or 2 years) taxa. While we processed reads to remove sequencing artifacts, some ephemeral ASVs may be a result of sequencing error (24). A majority of ASVs in both the small and large-sized fraction were ephemeral, as indicated by their markedly higher contribution to richness compared to persistent ASVs. Ephemeral taxa comprised 61%–80% of total comunity richness in 2016, 54%–63% in 2017, and 95%–96% in 2019. Within Dinoflagellata specifically for 2017, ephemeral and persistent ASVs contributed nearly equal to richness (49% and 50%, respectively). *Prochlorococcus* ephemeral ASVs accounted for 81% of richness in 2016, 45% in 2017, and 95% in 2019, while *Synechococcus* ephemeral ASVs accounted for 47%, 41%, and 93% in 2016, 2017, and 2019, respectively.

In contrast to their generally low contribution to total richness, persistent ASVs contributed substantially to total relative abundance (Fig. 2B). Persistent ASVs composed over 77% of the overall relative abundance each year across both eukaryotic and prokaryotic groups. Cyanobacteria had the highest contributions, with persistent *Synechococcus* and *Prochlorococcus* ASVs accounting for 93%–99% and 86%–99% of overall relative abundance, respectively. A mean Bray-Curtis dissimilarity distance of 0.75 (median 0.72) distinguished the relative abundance of ephemeral ASVs and the overall data set of ephemeral and persistent ASVs (Fig. 2C). On the other hand, persistent ASVs exhibited a mean value of 0.08 (median 0.09) when compared to the overall data set. Thus, persistent ASV distribution patterns more closely reflect the distribution patterns of the overall community, and we proceeded to use persistent taxa in subsequent network and modeling strategies.

### Phytoplankton distribution and covariation with biogeochemical trends

**Fig 3:**
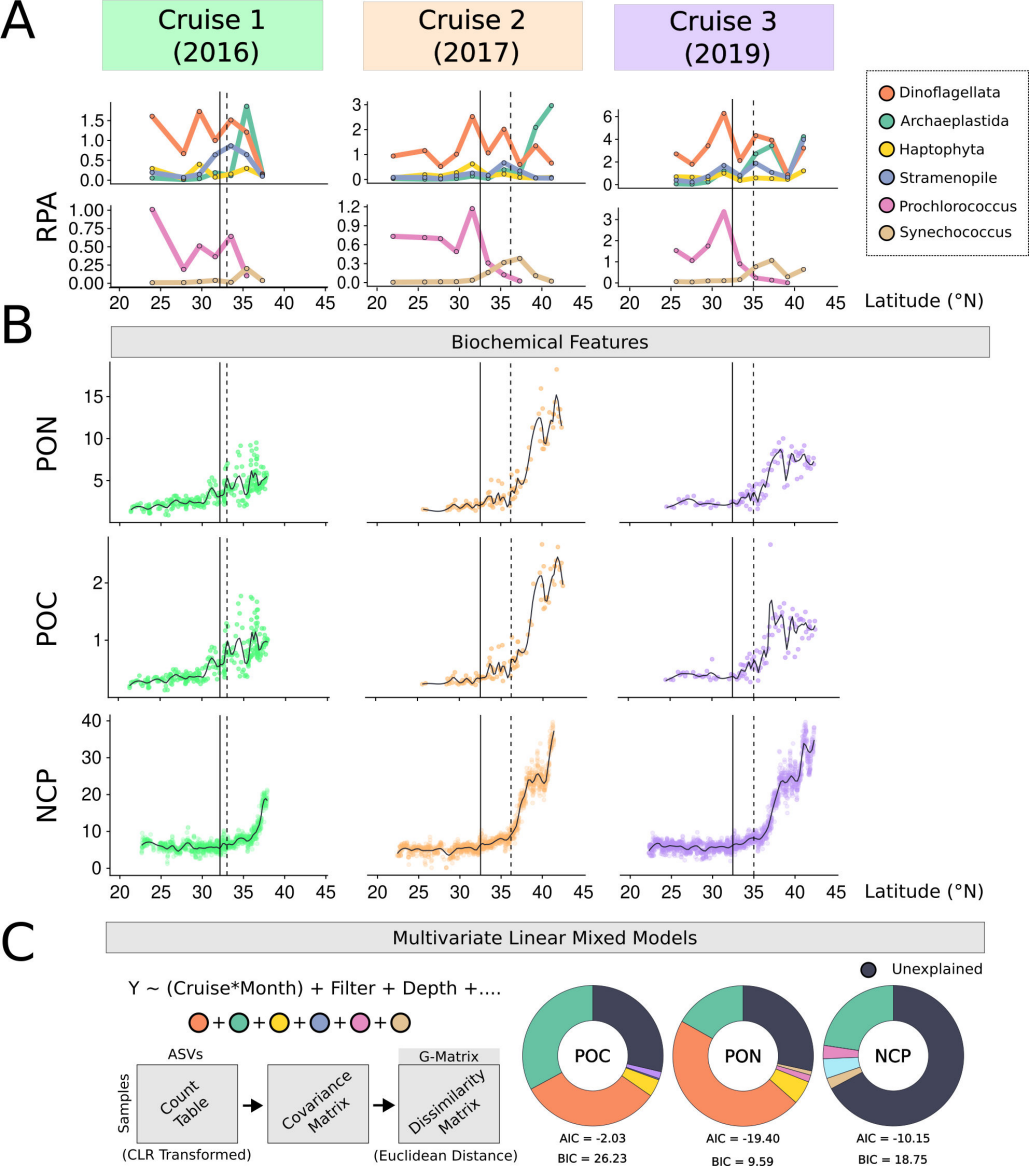
Microbial plankton contributions to spatiotemporal variations in biochemical parameters. (**A**) Relative percent abundance (RPA) of eukaryotic (top) and cyanobacterial (bottom) small-size fraction persistent phytoplankton groups across 2° latitude bins, highlighting changes in group dominance alongside biogeochemical patterns in panel **B**. Samples collected from all depths are considered. Solid and dashed lines denote the salinity front (isohaline 34.82) and the chlorophyll front (0.15 mg m^−3^), respectively. (**B**) Dot plots show latitudinal patterns of surface POC, PON, and NCP (0-15 m depth) from 2016 (green), 2017 (orange), and 2019 (purple) surveys. Black solid curve lines represent the fitted trend of the data. (**C**) MLMM formula (left) illustrating fixed effects (year interacting with month, filter size, and water column depth) and random variables (colored circles; phytoplankton-containing groups), with both size fractions included during modeling. Filter size categories include: 0.2 and 3 µm. Depth categories include 0–15 m, 45–75 m, and 90–125 m. Each response variable (Y) represents a biochemical feature (POC, PON, or NCP). Below, a schematic shows how G-matrices were built from ASVs grouped by each phytoplankton group. Donut plots (right) display the variance per biochemical feature explained by each phytoplankton group: Dinoflagellata (red), Archaeplastida (green), Haptophyta (yellow), Stramenopiles (blue), *Prochlorococcus* (orange), and *Synechococcus* (purple). Black segments indicate unexplained variance. Akaike information criterion (AIC) and Bayesian information criterion (BIC) scores below each plot assess model fit.

Relative percent abundance patterns of persistent phytoplankton-containing groups showed distinct biogeographic structuring across the transect (Fig. 3A), and we evaluated which groups were most closely aligned with previously published (18) concurrent measurements of NCP, POC, and PON (Fig. 3A through C; Fig. S5). Mean NCP, POC, and PON levels were highest within the NTZ (39.6 mmol O_2_ m^-2^ d^-1^, 18.2 μmol C L^-1^, and 2.7 μmol N L^-1^, respectively), followed by the STZ (10.7 mmol O_2_ m^-2^ d^-1^, 6.7 μmol C L^-1^, and 1.3 μmol N L^-1^, respectively), with lowest values in the NPSG (8.4 mmol O_2_ m^-2^ d^-1^, 2.7 μmol C L^-1^, and 0.4 μmol N L^-1^, respectively) (Fig. 3B). In addition, we leveraged previously published concurrent flow cytometry-based measurements of *Prochlorococcus* and *Synechococcus* biomass (25) to assess agreement with our 16S amplicon relative abundance data. ASVs captured the general latitudinal trends of both genera, with relatively strong correlation for *Prochlorococcus* (R = 0.81, *P* = 1.3 × 10^−7^) and *Synechococcus* (R = 0.59, *P* = 8.4 × 10^−4^) (Fig. S6).

We performed MLMM to assess the degree of covariation between persistent cyanobacterial and eukaryotic phytoplankton community structure and patterns in NCP, POC, and PON across the latitudinal transect (Fig. 3C). Dissimilarity-based G matrices, built from ASV-level data within each major phytoplankton-containing group, preserved fine-scale taxonomic resolution while capturing covariation with NCP, POC, and PON. Archaeplastida and Dinoflagellata were the top contributors to PON variability across the transect, explaining ~17% and 47% of the variance, respectively (AIC: –19.4; BIC: +9.6). Minimal covariation was found for Haptophyta (6%) and *Prochlorococcus* (2%), with 28% of the variance unexplained, potentially due to unmeasured environmental factors or stochastic processes. Archaeplastida and Dinoflagellata each explained ~32% of the POC variance (AIC: –2.0; BIC: +26.2). Haptophyta (4%) and *Synechococcus* (2%) contributed minimally, while 29% of POC patterns remained unexplained. Archaeplastida showed the highest covariation with NCP (~23%; AIC: –10.2; BIC: +18.8), followed by Stramenopiles (5%), *Prochlorococcus* (3%), and *Synechococcus* (3%), with 67% of NCP variance unexplained. While Archaeplastida distributions positively covaried with POC and PON values, Dinoflagellata had inverse relationships, suggesting the two groups play contrasting roles in POC and PON dynamics (Fig. 3B).

### Community-wide correlation of microbial plankton and biochemical variability

**Fig 4:**
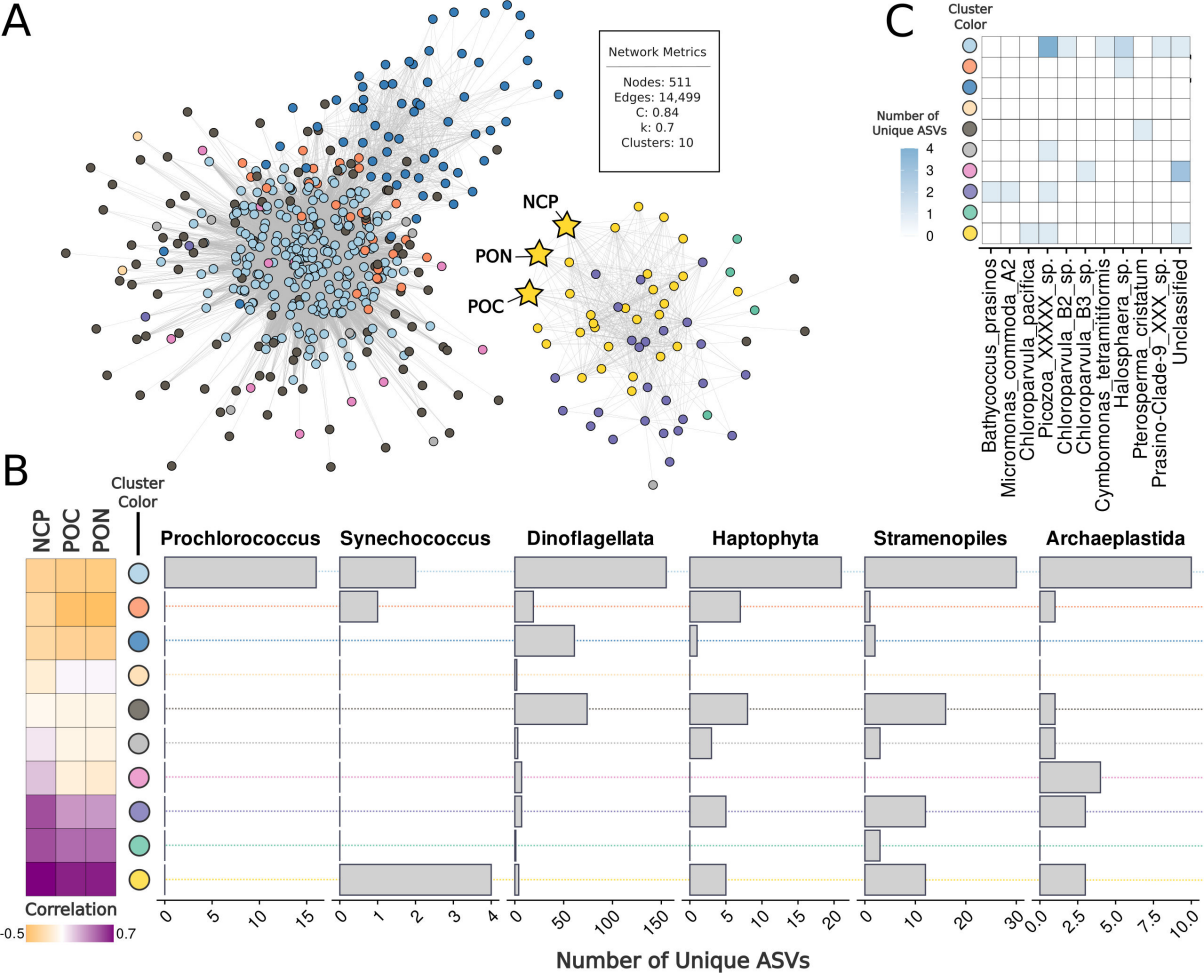
WGCNA of phytoplankton-containing communities and their association with biochemical measurements. (**A**) Network visualization showing ASV modules identified through WGCNA. Nodes are colored by module (cluster of ASVs) that exhibit coinciding spatial patterns across the transect. Nodes represent individual persistent ASVs, while edges represent the potential interactions existing between them. The biochemical measurement nodes NCP, POC, and PON are represented by yellow stars positioned among their most strongly correlated modules. The network consisted of 511 nodes and 14,499 edges, with a mean clustering coefficient (**C**) of 0.8 and network connectivity (k; density) of 0.7, organized into 10 clusters (see inset). Samples with available NCP, POC, and PON measurements were used in network construction. (**B**) Correlation plot (far left) and bar plots (right) showing module relationship to biochemical measurements and taxonomic composition of each cluster, respectively. Cluster colors are provided between the correlation and taxonomy plots and correspond to the WGCNA network in Panel **A**. Taxonomic bar plots show the number of unique ASVs within each major phytoplankton-containing group (*Prochlorococcus, Synechococcus,* Dinoflagellata, Haptophyta, Stramenopiles, and Archaeplastida). (**C**) A richness heatmap showing the ASV distribution across key Archaeplastida species within each network cluster in Panel **A**. Colors represent the number of unique ASVs for each species, from zero (white) to four (blue). Network cluster colors are provided to the right of the heatmap.

Weighted gene correlation network analysis (WGCNA) was used to determine whether specific persistent phytoplankton ASVs were correlated with the different environmental variables. The resulting network was organized into 10 distinct modules (clusters), with seven forming a densely connected central core and three (yellow, purple, and teal) forming smaller, more isolated clusters (Fig. 4A). NCP, POC, and PON were significantly associated with ASVs in the yellow (r = 0.7, 0.7, and 0.61, respectively; *P* < 0.001), purple (r = 0.5, 0.3, and 0.3; *P* < 0.001), and teal clusters (r = 0.5, 0.4, and 0.4; *P* < 0.001), with all three variables assigned to the yellow cluster (Fig. 4A). Regenerating the WGCNA network at varying powers (powers ranging from 1 to 8) confirmed that ASVs found within yellow and purple clusters remained consistent (Fig. S7). In contrast to yellow, purple, and teal clusters, several central core clusters exhibited predominately negative correlations with biogeochemical variables (r = –0.05 to –0.5, *P* < 0.001), with the sky blue and blue clusters being the most negatively correlated to NCP and red to POC and PON. Other core clusters showed weak positive or non-significant correlations (pink and light gray) with NCP (r = 0.2 and 0.07; *P* = 0.004 and 0.3, respectively), while the orange displayed weak positive, non-significant associations with POC and PON (r = 0.25 and 0.3; *P* = 0.65 and 0.7, respectively). These results suggest that the ASVs in the yellow, purple, and teal clusters may play an important role in shaping NCP, POC, and PON dynamics.

The yellow cluster contained 12 ASVs from Stramenopiles, 3 from Archaeplastida, 5 from Haptophyta, 4 from Dinoflagellata, and 4 from *Synechococcus* (Fig. 4B; Table S3). Archaeplastida members included *Chloroparvula pacifica*, an unclassified chlorophyte, and an unclassified Picozoa (Fig. 4C). Although classified within Archaeplastida, Picozoa are currently considered heterotrophic and distinct from chlorophytes (26). The cluster also featured other heterotrophic and mixotrophic protists, such as *Chrysochromulina* sp. (Haptophyta), MAST-1A, -1B, -1C, and -7A sp. (Stramenopiles), and a Parmales bolidophyte (Stramenopiles). Additional members were the parasitic dinoflagellate Syndiniales, haptophyte from *Phaeocystis*, silicoflagellate *Dictyocha speculum*, and stramenopile *Pseudochattonella*.

In the purple cluster, there were 12 ASVs from Stramenopiles, 3 from Archaeplastida, 5 from Haptophyta, and 7 from Dinoflagellata (Table S3). Archaeplastida members included *Bathycoccus prasinos, Micromonas commoda A2*, and another unclassified Picozoa (Fig. 4C). Like the yellow cluster, several ASVs belonged to *Chrysochromulina* sp., Syndiniales, and Parmales (*Triparma pacifica*). Other notable members included two haptophyte ASVs of *Phaeocystis* and stramenopiles such as *Pelagomonas calceolata, Triparma pacifica, Aureococcus anophagefferens* (pelagophytes), *Brockmanniella brockmannii* (diatom), and a member of the MAST-2D lineage. Finally, the teal cluster was small, only consisting of three ASVs (Table S3). These taxa included a member of the Syndiniales and three stramenopiles, one belonging to Naviculales, one to Thalassiosiraceae, and an unclassified species within the genus *Actinocyclus*.

### Key species co-occurrence patterns

**Fig 5:**
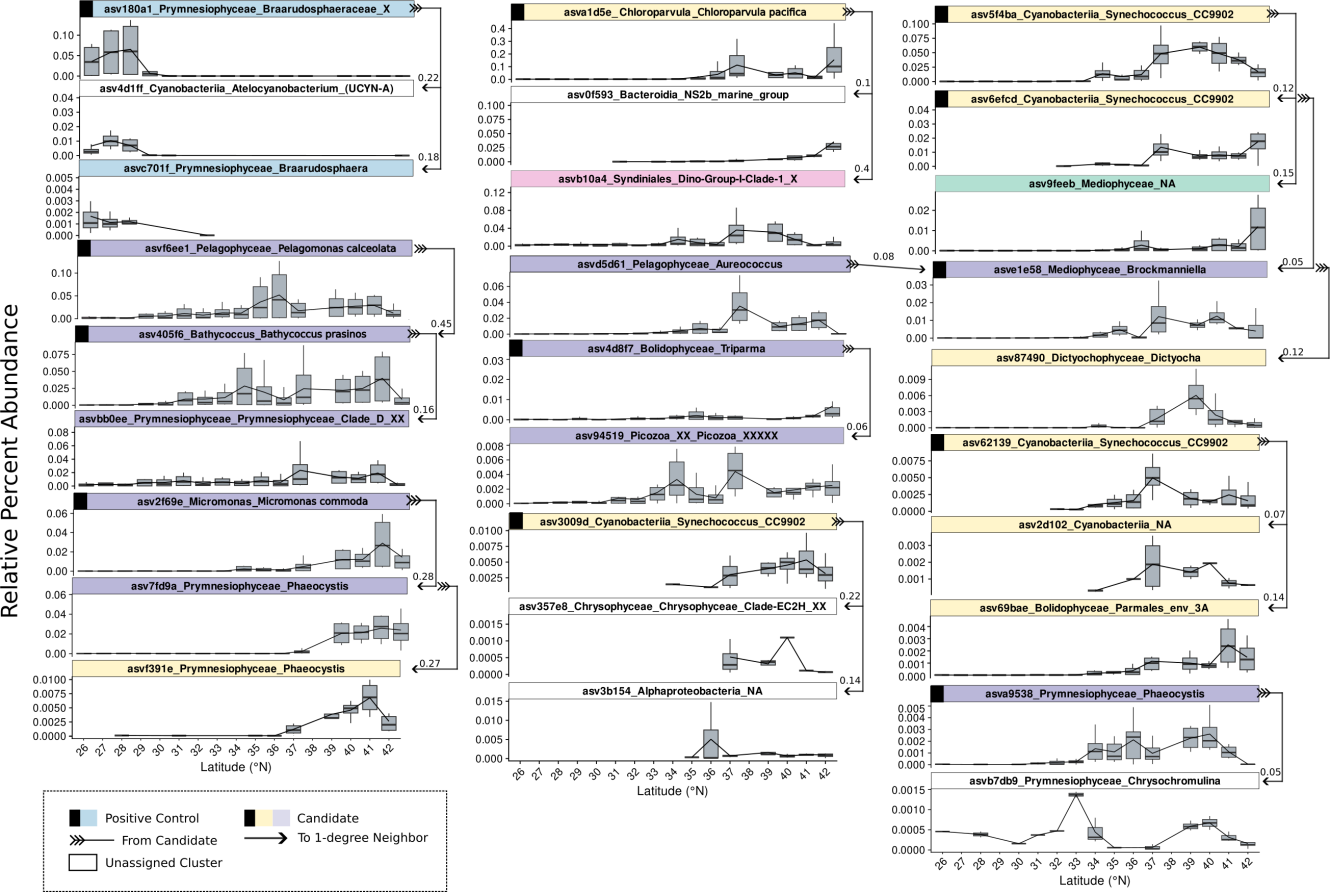
*In situ* distributions of yellow and purple cluster ASVs with their Top 1º Spiec-Easi neighbors. Relative percent abundance distributions in 2019 of yellow, purple, and teal ASVs (i.e., candidates) with full binomial species-level classifications (i.e., both genus and specific epithet), along with their top positively weighted first-degree neighbors from persistent phytoplankton and prokaryotic ASVs. Facet labels display each ASV’s unique ID, taxonomic classification, and WGCNA cluster assignment by color. Neighbors with no cluster assignment are shown in white. Arrows illustrate first-degree connections, originating from the candidate (feathered end) and pointing to its neighbor. Latitude is plotted on the x-axis, and relative percent abundance is shown on the y-axis. As internal validation, a known symbiosis between *Braarudosphaera* (the most abundant ASV from the family Braarudosphaeraceae assigned to the teal cluster) and the nitrogen-fixing cyanobacterium UCYN-A (*Candidatus Atelocyanobacterium thalassa*) was detected (top-left of the panel). Samples from all depths were used in Speic-Easi network construction.

We used the Spiec-Easi network analysis to identify taxa that displayed co-occurrence patterns and thus may potentially influence NCP, POC, and PON dynamics through metabolic interactions. The Spiec-Easi network included persistent ASVs from the six phytoplankton-containing groups, as well as all persistent 16S ASVs. This resulted in a moderately connected structure with 1,885 nodes and 37,350 edges averaging 40 neighbors per ASV (Fig. 5). Of these nodes, 1,006 were eukaryote ASVs, and 879 were prokaryote ASVs. As an internal validation, we examined whether a known association was recovered from this network: specifically, the symbiosis between *Braarudosphaera* and the nitrogen-fixing UCYN-A, shown to be an early-stage organelle, or nitroplast (27–29). Their co-occurrence within the network aligned with these prior studies (Fig. 5). Notably, the network was fully connected with no disconnected subnetworks. The *Chloroparvula pacifica* ASV from the WGCNA yellow cluster and the unclassified Picozoa ASV from the purple cluster were among the ASVs with highest connectivity (i.e., out-degree) for eukaryotes (Fig. 5; Table S4), while ASVs from Alphaproteobacteria, Bacteroidia, and Actinobacteria contained the highest out-degree connections within the heterotrophic bacterial community (Table S4).

We analyzed weights and cluster assignments for first-degree (1°) neighbors (i.e., ASVs connected directly by one network edge) for ASVs within yellow, purple, and teal WGCNA clusters that positively correlated to NCP, POC, and PON measurements (Fig. S8). Association weights ranged from –0.15 to 0.46 (mean = 0.03), with largest absolute weight values indicating strongest associations. Positive associations spanned 9.9 × 10⁻⁶ to 0.46, while negative associations ranged from –0.15 to –1.2 × 10^⁻4^ (Fig. S8A). Positively weighted 1° neighbors tended to belong to the same yellow, purple, and teal WGCNA clusters (Fig. S8B). In contrast, negatively weighted neighbors were primarily housed in WGCNA clusters that negatively correlated with NCP, POC, and PON measurements, such as the red, blue, and sky blue clusters (Fig. 4B and 8B). Transect relative abundance profiles for positive neighbor connections to yellow and purple candidates with complete binomial species classifications (i.e., genus and species epithet) are highlighted in Fig. 5. Teal members did not serve as candidates themselves but instead appeared as associates of yellow or purple members. We specifically focused on neighbors with the highest connectivity (i.e., weight). All positive yellow and purple WGCNA neighbor-candidate connections are in Table S5.

From the yellow cluster, the *Chloroparvula pacifica* ASV showed highest positive connection to a Syndiniales Dino-Group-1-Clade-1 ASV (weight = 0.37) among eukaryotic associates and an NS2B Bacteroidia NS2b Marine Group ASV (weight = 0.1) among prokaryotic associates (Fig. 5). Four *Synechococcus sp*. CC9902 ASVs were also found within the yellow cluster. One with the identifier ‘5f4ba’ exhibited the highest connection with an unknown diatom species from Mediophyceae (0.15) from the teal cluster and with another yellow-cluster *Synechococcus* sp. CC9902 ASV (0.12). This second *Synechococcus* ASV “6efcd” showed its highest-weighted connection to a purple-cluster diatom *Brockmanniella brockmannii* (0.05). The remaining two *Synechococcus* sp. CC9902 ASVs showed the highest-weighted eukaryotic connections to an unclassified Chrysophyceae ASV (0.22) and a yellow-cluster Parmales ASV (0.14), and prokaryotic associations to an unclassified Alphaproteobacteria (0.14) and Cyanobacteria (0.07), respectively (Fig. 5). The same Parmales ASV was also connected to a MAST-1A ASV, also from the yellow cluster (Table S5). We also found one *Phaeocystis* ASV connected to a yellow-cluster *Chrysochromulina* ASV (0.04) (Fig. 5), which shared an edge with the same MAST-1A ASV (0.18) mentioned above (Table S5). This MAST-1A ASV itself neighbored two additional yellow-cluster stramenopiles: a Parmales_env_3A ASV (0.1) and a MAST-7A ASV (0.05). No positive prokaryotic neighbors were detected for the mentioned Parmales, *Phaeocystis*, *Chrysochromulina,* and MAST ASVs.

For purple-cluster Archaeplastida members, the *Bathycoccus prasinos* ASV showed the highest positively weighted connection with another purple-cluster member *Prymnesiophyceae* Clade D ASV (0.16) (Fig. 5). One *Phaeocystis* ASV itself had no prokaryotic associates but connected to the *B. prasinos* ASV (0.45). For *Micromonas commoda*, top associations included the purple-cluster haptophyte *Pelagomonas pouchetii* (0.28). *Triparma pacifica* showed the highest eukaryotic connection to a purple cluster, unclassified Picozoa ASV (0.06), and lacked prokaryotic associations. The purple-cluster diatom *B. brockmannii* was associated with *Dityocha speculum* (0.12) and was an associate to *Aureococcus anophagefferens* (0.08), both of which belonged to the purple cluster, but showed no positive links to prokaryotic ASVs. Finally, another *Phaeocystis* ASV*,* classified as *P. pouchetii,* exhibited its highest-weighted connection with *P. antarctica* in the yellow cluster (0.27). Due to limited taxonomic resolution for the *Phaeocystis genus* with the 18S V4 primer, we note these species-level assignments are uncertain (30, 31).

## DISCUSSION

Our spatiotemporal study revealed distinct shifts in phytoplankton cell size composition and community structure across the natural physio-chemical gradient of the NPTZ (Fig. 1C; Fig. S2 and S3). In the southern region of the transect, *Prochlorococcus* (pico-plankton) and Dinoflagellata (nano- and micro-plankton) relative abundances were the highest but then declined just north of the NPSG, a trend consistent with previous studies that analyzed biomass estimates and metatranscriptomic data of major taxonomic groups (17, 23). Within the more productive NTZ, *Synechococcus* (pico-), Archaeplastida (pico-), and Stramenopiles (pico- to micro-plankton) increased in relative percent abundances. Our study revealed that persistent ASVs (those detected in all 3 years) dominated the total relative abundance and reflected overall community patterns across all cruises (Fig. 2). This suggests a stable “core” set of taxa that control community structure and biogeochemistry in the NPTZ. While this study leverages compositional amplicon data, which reflect relative sequence abundances that limit our ability to infer absolute organismal abundance (32) and may also be influenced by variable rRNA gene copy numbers (33) (Fig. S6), we were able to link compositional shifts of core taxa with concurrent biochemical trends. In particular, we identified specific plankton taxa, particularly pico-sized groups, that likely shape elemental stoichiometry and ecosystem productivity. Furthermore, comparing another network approach more tailored for compositional data (Spiec-Easi), rather than phenotype-trait relationships (WGCNA), revealed shared overlap and insight into putative trophic interactions to explore further.

### Microbial plankton composition and stoichiometry

Stoichiometry measurements from the North Pacific have demonstrated that the NPSG exhibits elevated C:N ratios relative to Redfield, whereas the NPTZ typically display lower ratios (34). This study builds on the Redfield Ratio theory (35) by examining how phytoplankton community composition relates to POC and PON levels across a latitudinal gradient. While the Redfield Ratio (106:16:1) has historically been used to describe marine particulate matter composition, numerous studies have demonstrated that elemental stoichiometry is highly variable across oceanic regions, particularly in response to environmental gradients (34–36). Whether these regional patterns are primarily driven by taxonomic composition or environmental constraints is debated. Some studies argue that oceanographic conditions dictate stoichiometric variability (36, 37), while others suggest that taxonomic differences in macromolecular allocation play a critical role (38, 39).

Previous work has shown that dinoflagellates exhibit higher C:N ratios, likely reflecting an increased allocation to carbon-enriched macromolecules, such as energy-storage lipids (39). This trait likely enhances their ability to thrive in oligotrophic environments where nitrogen availability is limited (Fig. 3B), a pattern consistent with their high proportion of ASV reads in the NPSG (Fig. 3A). Conversely, Archaeplastida display lower C:N ratios compared to Dinophyceae members (39), suggesting a more balanced allocation of carbon and nitrogen. This stoichiometric balance likely contributes to the success and increase in the relative abundance of Archaeplastida taxa in nitrogen-enriched regions of the NPTZ (Fig. 1C). Our findings align with previous arguments by (39) and (38), who proposed that differences in elemental stoichiometry among phytoplankton groups arise from evolutionary trade-offs in macromolecular allocation, particularly under varying nutrient regimes, which in turn shape their biogeographic distributions (38, 39). We observed distinct shifts in community structure corresponding to changes in POC and PON, particularly for Archaeplastida and Dinoflagellata, that support this framework (Fig. 3).

### Chlorophyta populations and NCP

Archaeplastida were consistently enriched in the NTZ (Fig. 1C) and dominated by a few persistent ASVs (Fig. 2A and B). Members of Archaeplastida exhibited strong covariation with POC, PON, and NCP based on MLMM (Fig. 3C), and the WGCNA identified specific chlorophyte ASVs closely aligned with NCP, such as *B. prasinos, C. pacifica*, and *M. commoda A2* (Fig. 4C). The picoeukaryotes *Bathycoccus* and *Micromonas* dominate nutrient-rich coastal regions and sporadically bloom under favorable conditions (6, 40–42). *Bathycoccus* has also been observed in deeper epipelagic zones where light and nutrient availability are more limited (43), and *Micromonas* is an important member of eukaryotic picophytoplankton communities in the Arctic, Atlantic temperate waters, and various coastal environments (44). Our findings further highlight the importance of chlorophyte picoeukaryotes to open-ocean carbon and nutrient cycling.

Recent advances in molecular-based taxonomic resolution have expanded our understanding of open-ocean Chlorophyta members, particularly through the identification of chlorophyte clade VII, which dominates surface photic zones in oligotrophic marine environments (41, 45). This clade was recently revised with the formal description of two novel classes: Picocystophyceae and Chloropicophyceae, the latter of which contains the newly described genera *Chloropicon* and *Chloroparvula*. Notably, we identified *C. pacifica* as a persistent ASV in the NTZ that exhibited strong covariation with NCP measurements, suggesting its role in regional carbon dynamics (Fig. 4C). Our findings highlight chlorophytes as key players in regional carbon and nitrogen dynamics and a need for continued investigation of recently resolved Chlorophyta picophytoplankton groups.

### Protistan mixotrophs and grazers

We identified several mixotrophic and heterotrophic protist ASVs coinciding with picophytoplankton communities based on WGCNA clustering and Spiec-Easi-derived associations (Fig. 4C and 5). Unclassified Picozoa were detected in the WGCNA yellow and purple clusters, which also included *Synechococcus* and autotrophic chlorophytes. Picozoa are currently uncultured members of the Archaeplastida, deemed to lack plastids and presumed to have a heterotrophic lifestyle (26, 46). Genomic evidence has revealed viral-origin DNA within their cells, raising the possibility of viral particle ingestion (26, 47). There are reports of DNA viruses infecting marine chlorophytes, particularly *Bathycoccus* and *Micromonas* (48–51), and *Synechococcus* (52, 53). While our data set did not assess viral DNA, the observed Picozoa-picophytoplankton co-occurrence suggests a plausible ecological scenario in which Picozoa indirectly influence these members by targeted viral grazing (53) of those viruses infecting *Synechococcus* or picoeukaryotic phytoplankton or grazing of microbial antagonists.

We also observed several associations that may reflect direct trophic interactions, such as grazing by larger protists. Four *Synechococcus* ASVs within the WGCNA yellow cluster co-occurred with members of heterotrophic MAST lineages (MAST-1 and MAST-7), mixotrophic *Chrysochromulina* sp. (Haptophyta), and the Parmales group, with the trophic modes of these protists cross-referenced against curated databases (54, 55) and prior studies (50, 56). The SpiecEasi network additionally detected an association between Parmales and *Synechococcus* ASVs (Fig. 5). Prior studies have demonstrated that *Synechococcus* populations are strongly influenced by top-down controls from plankton grazers, including both heterotrophic and mixotrophic protists (57, 58). For example, recent genomic analyses suggest that Parmales may exhibit phago-mixotrophy, combining photosynthesis with the ingestion of prey to better thrive under nutrient-limited conditions (50). Additionally, a past study in the East China Sea during the summer season demonstrated that MAST grazers strongly correlated with *Synechococcus* populations, with experimental validation confirming their ingestion of *Synechococcus* in culture (59). While MAST members were not the strongest eukaryotic neighbors to any *Synechococcus* ASVs, they exhibited the strongest weighted association with purple cluster members *Chrysochromulina* and Parmales, which in turn connected to *Synechococcus*, suggesting an indirect influence on its population dynamics. These patterns highlight the complexity of picoplankton interactions within the NPTZ and underscore the need for future studies to disentangle the nature and directionality of these putative associations.

### Conclusion

Microbial “neighborhoods” shape ecosystem dynamics through ecological associations, wherein metabolic coupling, niche construction, and trophic interactions form an emergent, self-sustaining unit that impacts broader ecosystem dynamics (60–62). Our integrated modeling and co-occurrence network strategies proved useful to identify key neighborhood members in the NPTZ. Unlike direct interaction studies, network-based and systematic modeling approaches capture emergent patterns of microbial co-existence found in nature, revealing hidden ecological roles that shape elemental biogeochemical cycles. We recapitulated the well-documented association between *Braarudosphaera* and its nitrogen-fixing symbiont/organelle UCYN-A (27, 63), a relationship currently considered obligate. We also identified a chlorophyte-centric neighborhood that likely underlies enhanced NCP and altered stoichiometry in the NPTZ and, interestingly, may be directly or indirectly controlled by grazing. These observed associations highlight the use of network analysis to detect a range of complex associations, some of which may operate through direct and indirect ecological mechanisms. As the field of microbial ecology advances, statistical modeling and network-based co-occurrence analyses will be essential for unraveling the complexity of microbial interactions, offering a systems-level approach to identifying both emergent symbiotic relationships and broader ecosystem dynamics.

## MATERIALS AND METHODS

### Amplicon collection and sequencing

Seawater samples for 16S and 18S rRNA gene sequencing were collected during three cruises: Gradients 1 (April 19–May 5, 2016; 23°N to 37°N), Gradients 2 (May 25–June 13, 2017; 21°N to 41°N), and Gradients 3 (April 9–30, 2019; 26°N to 42°N), all along the ~158°W longitude transect. A total of 48 samples were collected in 2016, 72 in 2017, and 192 in 2019. Samples were collected via CTD rosettes or shipboard surface-intake systems. Sampling depth ranged from 0 to 110 m in the 2016 survey, 12–15 m for 2017, and 0–125 m for 2019. For each sampling site with a unique latitude, depth, and time, 1–3 replicates were collected per size fraction (Data set S1). Salinity and chlorophyll fronts delineating NPTZ regions were defined following Juranek et al. (18).

A peristaltic pump was used to sequentially filter cells onto 3.0-μm pore size 25 mm polyethersulfone membranes (Sterlitech, Kent) and 0.2-μm pore size 25 mm Supor membranes (Pall Corporation, New York). Filters were immediately flash frozen in liquid nitrogen and subsequently stored at −80°C until processing. Genomic DNA was extracted following protocols from Gradoville et al. (21). In brief, samples were processed using DNeasy Plant Mini Kits following manufacturer protocols (Qiagen, Venlo, the Netherlands). Additional steps included three freeze-thaw cycles, 2 min of bead-beating, and a Proteinase K treatment, as described by Moisander et al. (64). The V4 region of the 16S rRNA gene was amplified using primers 515F/806R (65), and the V4–V5 region of 18S rRNA gene using 566F/1,200R (66), of which only the 566F primer was used (see the supplemental methods). These amplified genetic loci regions were then barcoded, quantified, pooled, and sequenced using 250 bp paired-end reads on an Illumina MiSeq.

### Amplicon processing and community analysis

ASVs were processed using QIIME2 (67). Sequences were quality-controlled, trimmed, merged, and annotated as described in the supplemental methods. ASV counts were rarefied (Table S6) prior to calculating relative percent abundance values. 16S and 18S ASVs were annotated against the SILVA database (68) and PR2 database v5.1 (69, 70), respectively, and further categorized as either persistent (present in all three cruises) or ephemeral (present in only one or two cruises). NMDS was performed using *vegan* v2.6-4 (71). Details can be found in the supplemental methods.

### Physiochemical, biochemical, and biomass data

Physiochemical measurements (seawater surface temperature and salinity), biochemical measurements (POC, PON, and NCP), and cyanobacterial biomass estimates were obtained for the Simons Collaborative Marine Atlas Project (25), with data links provided in the supplemental methods. Amplicon samples were paired with corresponding CMAP data (Fig. S5) as described in the supplemental methods.

### Multi-level mixed modeling and network analysis

MLMM was performed within the *sommer* package v4.3.3 (72). Weighted gene co-expression network analysis (WGCNA) v1.72-5 (73) was applied to persistent ASVs from phytoplankton-containing taxa and Box-Cox–transformed NCP, POC, and PON values to identify ASV modules correlated to each biochemical variable. Details can be found in the supplemental methods. Sparse Inverse Covariance Estimation for Ecological Association Inference (Spiec-Easi) v1.1.0 (74) was used to infer direct associations between specific persistent eukaryotic and prokaryotic ASVs (Data set S2). Details can be found in the supplemental methods.

## Data Availability

Raw 16S and 18S rRNA gene amplicon data are deposited to the NCBI Sequence Read Archive (SRA) under BioProject accession number PRJNA1302492. Processed ASV count tables, taxonomic classifications, and sample metadata are archived on Zenodo. Dataframes are also available via the Simons Collaborative Marine Atlas Project (CMAP). All analysis scripts used for QIIME2, dataframe pre-processing, statistical analysis, and figure generation are available on Github. Processed dataframes are available at https://zenodo.org/records/17288024. The CMAP Cruise 1 dataset is available at https://simonscmap.com/catalog/datasets/G1_ASVs, the CMAP Cruise 2 dataset is available at https://simonscmap.com/catalog/datasets/G2_ASVs, the CMAP Cruise 3 dataset is available at https://simonscmap.com/catalog/datasets/G3_ASVs, and all analysis scripts are available on GitHub at https://github.com/rkeyMicrobe/picoGrads2025.

## SUPPLEMENTAL MATERIAL

Data set S1 msystems.00801-25-s0001.csv Sample site metadata.

Data set S2 msystems.00801-25-s0002.csv Spiec-Easi associations.

Supplemental File msystems.00801-25-s0003.pdf Supplemental figures, tables, and methods.

ASM does not own the copyrights to Supplemental Material that may be linked to, or accessed through, an article. The authors have granted ASM a non-exclusive, world-wide license to publish the Supplemental Material files. Please contact the corresponding author directly for reuse.
