# Supplementary material for: Picophytoplankton implicated in productivity and biogeochemistry in the North Pacific Transition Zone
Source: mSystems. 2026 Jan 16;11(2):e00801-25. doi: 10.1128/msystems.00801-25 (PMC12911361; doi:10.1128/msystems.00801-25)
Supplement: Supplemental File — Supplemental figures, tables, and methods. [file msystems.00801-25-s0003.pdf]

SUPPLEMENTAL FIGURES

S1: Sample Site Depth Profiles for Each Cruise
S2: Eukaryotic 18S Spatial and Temporal Patterns across Yearly Transects
S3: Prokaryotic 16S Spatial and Temporal Patterns across Yearly Transects
S4: Relative Percent Abundance of Major Taxonomic Groups Across Filter Sizes and Regions
S5: Temporal and spatial offsets after integrating amplicon, POC, PON, and NCP datasets
S6: Amplicon and biomass comparison of *Prochlorococcus* and *Synechococcus* during the 2019 G3 cruise.
S7: WGCNA soft-threshold power and module clustering of persistent phytoplankton Biochemical Variables.
S8: Spiec-Easi-Derived Weight Range and Cluster Assignments for 1° Spiec-Easi Neighbors of Purple and Yellow Candidates

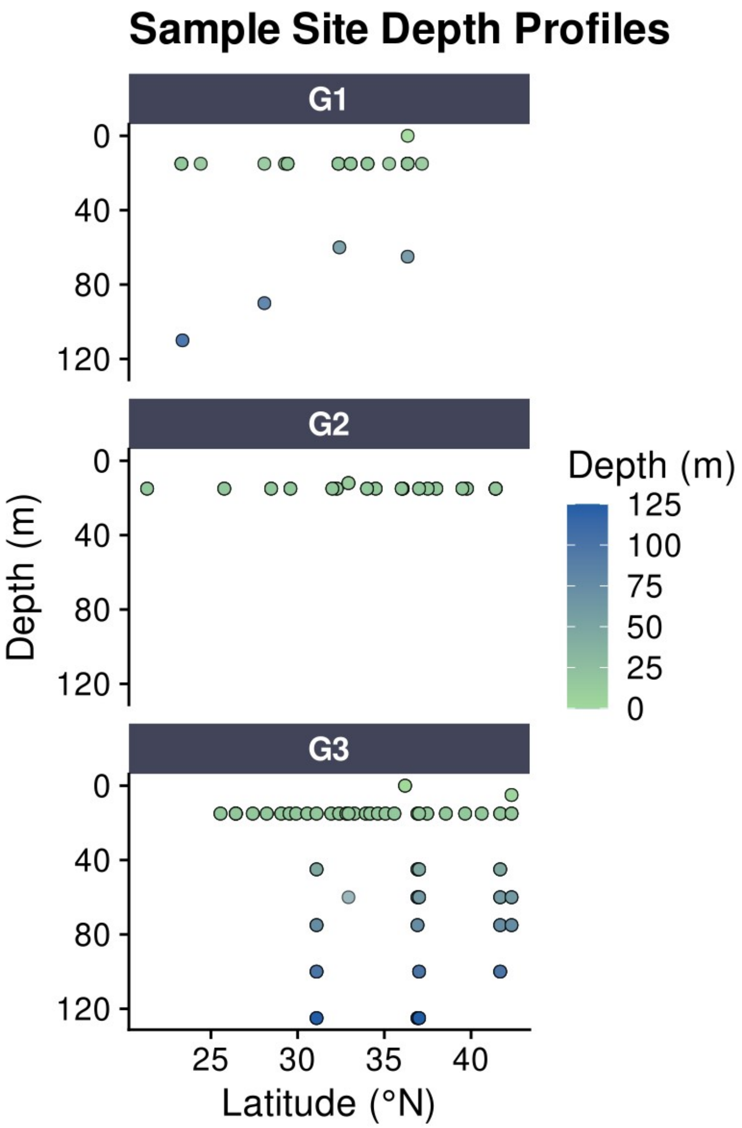

Fig. S1. Sample Site Depth Profiles for Each Cruise
Sampling depths (y-axis) and latitudes (x-axis) of all amplicon samples collected during Gradients 1 (G1), Gradients 2 (G2), and Gradients 3 (G3) cruises. Each point represents a unique sampling site that includes both size fractions (0.2–3 μm and >3 μm). Points are colored by collection depth, with light green indicating surface waters and dark blue indicating deeper samples. Samples were collected using either CTD casts or the ship's surface-intake systems. See Dataset S1 for sample metadata.

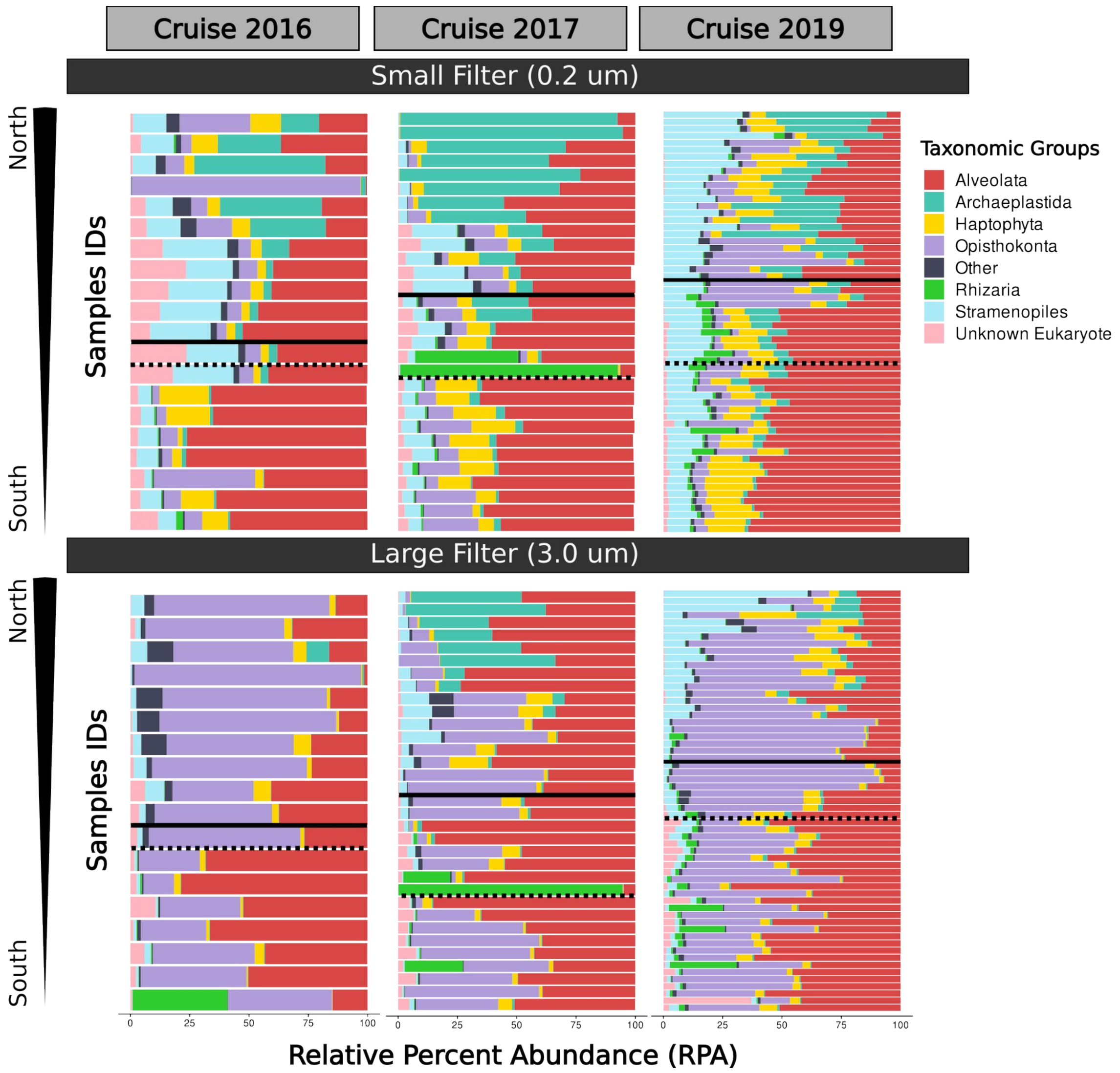

Fig. S2. Eukaryotic 18S Spatial and Temporal Patterns across Yearly Transects. Relative percent abundance bar charts for small size fraction samples (top) and large size fraction samples (bottom). Samples are arranged by latitude on the y-axis, and the x-axis displays the total relative percent abundance of each major taxonomic group at the phylum level. 'Unknown Eukaryote' represents eukaryotic ASVs with unknown taxonomic classification at the phylum level. The 'Other' category includes taxonomic groups representing less than 1% of total relative percent abundance per cruise year. The salinity front is denoted by a dotted black line, while the chlorophyll front is denoted by a solid black line.

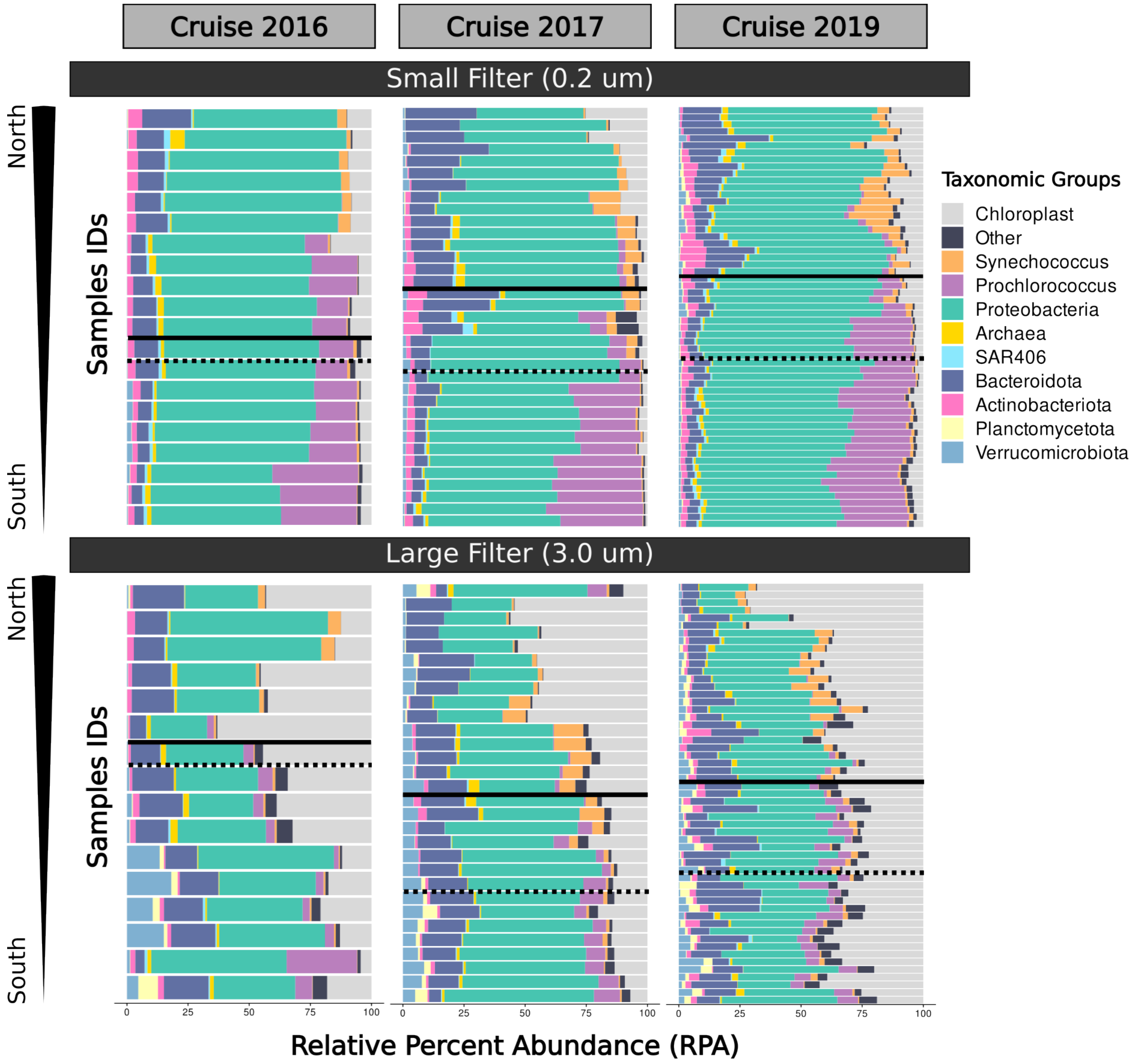

Fig. S3. Prokaryotic 16S Spatial and Temporal Patterns across Yearly Transects. Relative percent abundance bar charts for small size fraction samples (top) and large size fraction samples (bottom). Samples are arranged by latitude on the y-axis. The x-axis shows total relative percent abundance of each major taxonomic group at the phylum level. The 'Prochlorococcus' and 'Synechococcus' categories represent ASVs classified under the respective genera *Prochlorococcus* and *Synechococcus*. The 'Archaea' category groups all ASVs with the domain classification Archaea. The 'Chloroplast' category represents prokaryotic ASVs classified as Chloroplast at the class level. The "Other" category encompasses taxonomic groups representing less than 1% of total relative percent abundance per cruise year, including other cyanobacteria not classified within the *Prochlorococcus* and *Synechococcus* categories. The salinity front is denoted by a dotted black line, while the chlorophyll front is denoted by a solid black line.

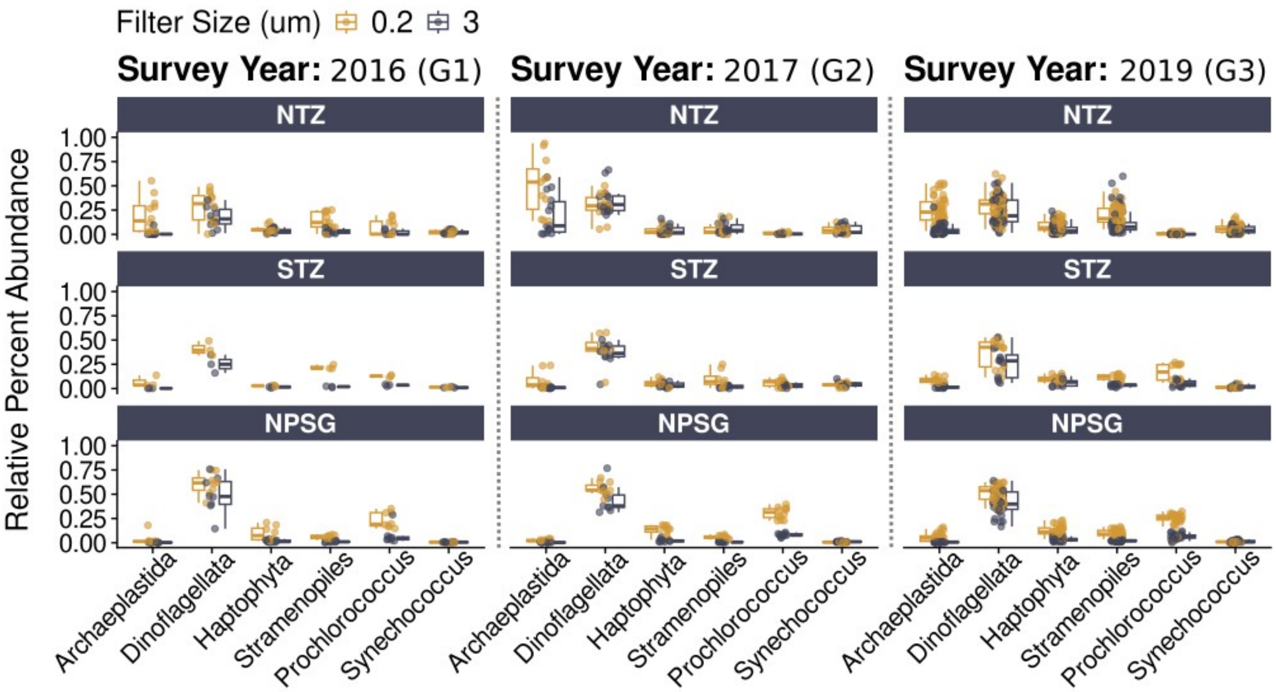

Fig. S4. Relative Percent Abundance of Major Taxonomic Groups Across Filter Sizes and Regions. Relative percent abundance of major phytoplankton-containing groups across three regions: NTZ (Northern Transition Zone), STZ (Southern Transition Zone), and NPSG (North Pacific Subtropical Gyre) during three survey years (G1 2016; G2 2017; G3 2019). Values for prokaryotic (*Prochlorococcus* and *Synechococcus*) and eukaryotic (Archaeplastida, Dinoflagellata, Haptophyta, Stramenopiles) relative percent abundances were calculated relative to their corresponding community. Boxplots show the mean and spread of relative percent abundance across samples, with individual samples points plotted adjacent to their respective box plot. Filter size fractions are indicated by gold (0.2 µm) and gray (3 µm). Columns represent each region across the three survey years. Samples from all depths were considered.

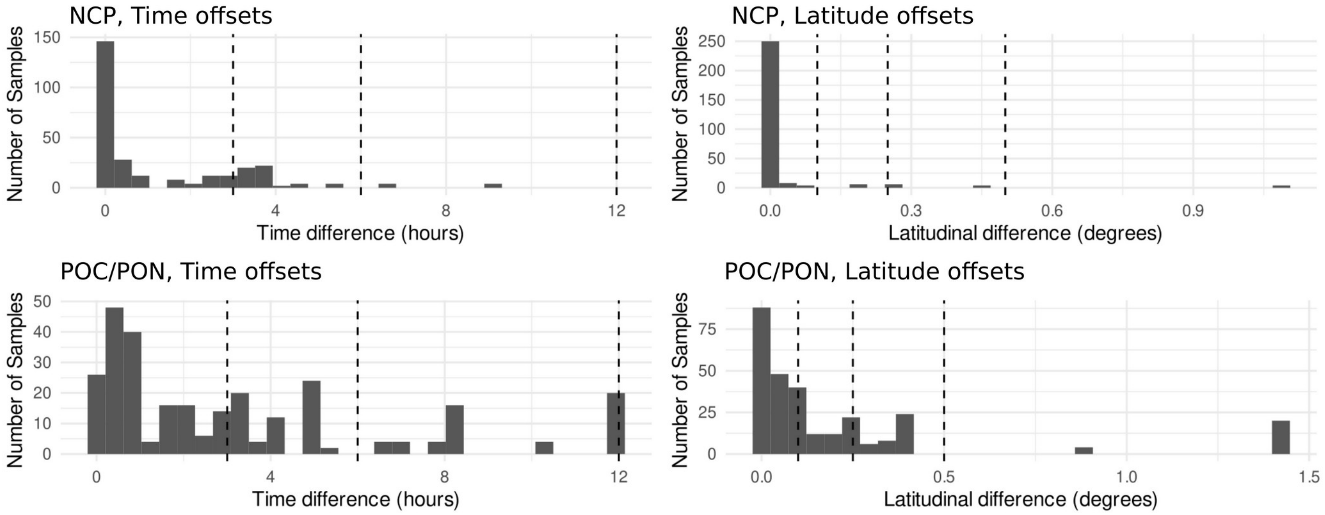

Fig S5. Temporal and spatial offsets after integrating amplicon, POC, PON, and NCP datasets. Distributions of temporal (hours; left) and latitudinal (°N; right) offsets between amplicon samples and paired NCP (top) or POC/PON (bottom) measurements. Amplicon samples were matched to biogeochemical data using a maximum cutoff of 12 hours and 0.5° latitude. Dashed vertical lines mark thresholds of 3, 6, and 12 hours (time) or 0.1°, 0.25°, and 0.5° (latitude). Offsets were highly concentrated near zero, with most matches within ~0–4 hours and <0.2° latitude windows. O_2/Ar-derived NCP measurements reflecting mixed-layer O_2 turnover over ~10–15 days (Juranek et al., 2020) and is unlikely to be biased by temporal offsets.

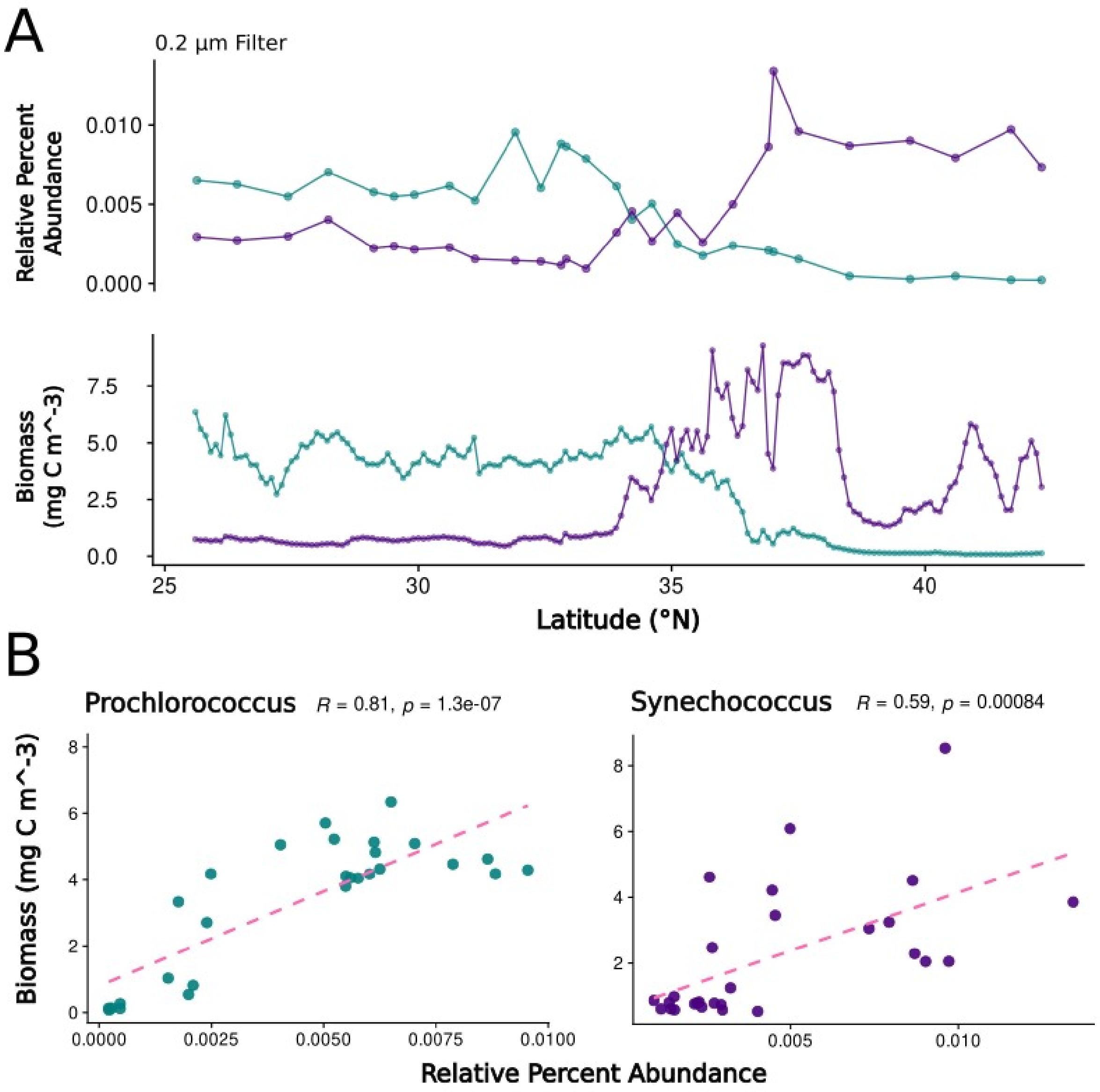

Fig S6. **Amplicon and biomass comparison of *Prochlorococcus* and *Synechococcus* during the 2019 G3 cruise. (A)** Relative percent abundance (top) and corresponding SeaFlow-derived biomass estimates (mg C m^{-3}; bottom) across latitude for *Prochlorococcus* (teal) and *Synechococcus* (purple). Amplicon data are from the 0.2 µm size fraction across all depths and are averaged by every 0.1° of latitude. **(B)** Scatterplots showing relationships between relative percent abundance (x-axis) and biomass (y-axis). Dashed lines indicate linear regression fits; Pearson's correlation coefficients (R) and p-values are shown for each panel.

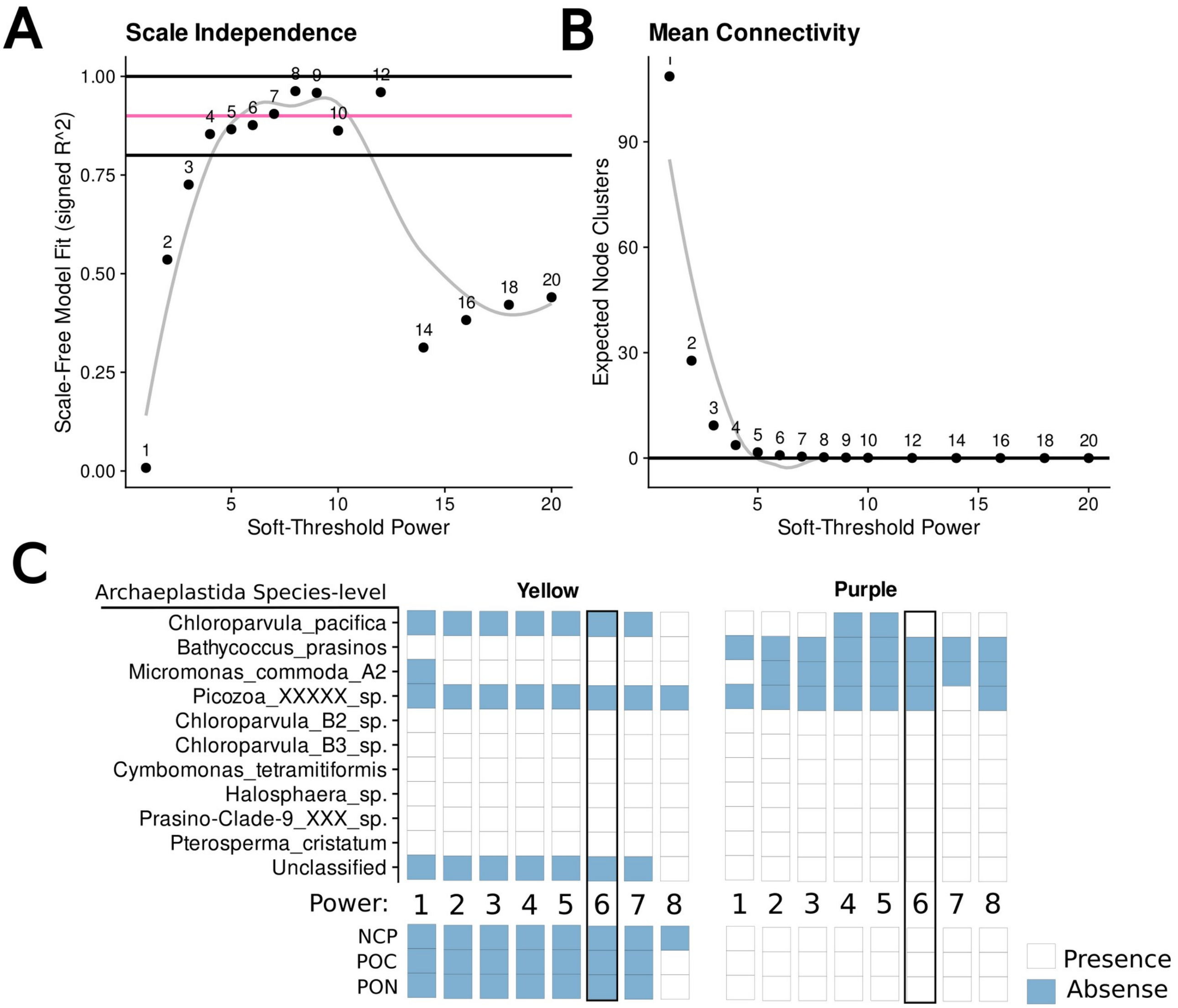

Fig S7. WGCNA soft-threshold power and module clustering of persistent phytoplankton Biochemical Variables. (A) A scale independence plot showing the fit of the network to a scale-free topology across a range of soft-thresholding powers. The y-axis shows the signed R^2, which indicates the degree to which the network fits a scale-free topology. A power of 6 was chosen as it provides a high R^2 value (near 0.9 represented by the pink line). Solid black lines denote 0.8 and 1.0 R^2 values. **(B)** Mean connectivity plot showing the number of expected node clusters, or modules for the different soft-threshold powers. A power of 6 was selected as it provides a good balance between high model fit found in Panel A and low mean connectivity that is over zero. **(C)** Heatmap of Archaeplastida species-level ASVs showing their presence (blue) or absence (white) in the clusters associated with biochemical variables (POC, PON, and NCP) across WGCNA soft-thresholding powers ranging from 1 to 8. If the power is too high, the network becomes too sparse, making it harder to detect meaningful connections. If it is too low, weak or random correlations may be kept, making the patterns less reliable. The chosen power reflects a balance between filtering noise and preserving biologically meaningful patterns.

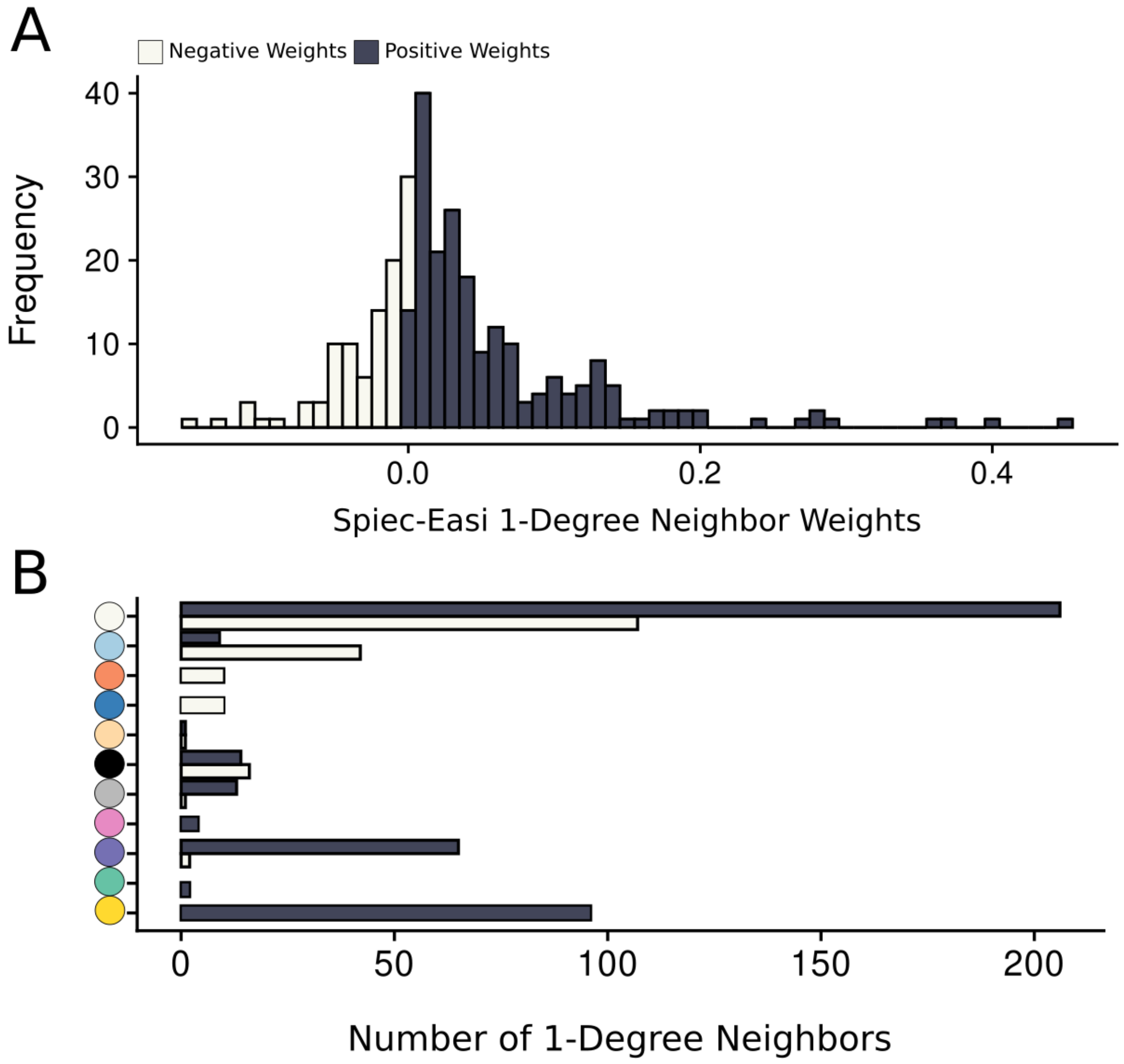

Fig S8: Spiec-Easi-Derived Weight Range and Cluster Assignments for 1° Spiec-Easi Neighbors of Purple and Yellow Candidates

(A) Distribution of Spiec-Easi first-degree neighbor weights for ASVs (i.e. Candidates) in the yellow and purple WGCNA clusters. White bars indicate negative weights, while green bars represent positive weights. **(B)** Spiec-Easi first-degree neighbors for candidates found within WGCNA clusters grouped based on neighbor's WGCNA cluster assignments. The same bar color theme as Panel A is applied. The 'No Assignment' cluster (white) represents ASVs excluded from WGCNA network construction due to quality control filtering for sparsity and heterotrophic bacteria.

SUPPLEMENTAL TABLES

S1: ANOSIM Results Across Yearly Surveys
S2: EnvFit NMDS Correlations on Group Relative Percent Abundance
S3: Taxonomic Composition of Yellow and Purple WGCNA Clusters
S4: Top 5 Out-degree ASVs for Prokaryotes (16S) and Eukaryotes (18S)
S5: Yellow and Purple Candidate Connections from Spiec-Easi Network
S6: Rarefaction Thresholds for 18S and 16S Amplicon Sequencing Across the Yearly Surveys

Table S1. ANOSIM Results Across Yearly Surveys. Results from an ANOSIM analysis performed on samples across the three yearly surveys (G1 = Cruise 2016, G2 = Cruise 2017, G3 = Cruise 2019). Both R values and p-values for 'Filter', 'Region', and ‘Depth’ variables were evaluated. Higher R values indicate greater separation of community composition between groups, with values near zero indicating little to no separation. Survey observations are colored to reflect the chosen color palette used in other figures. Cruise 2016 is in green, 2017 in orange, and 2019 in purple. P-values ≤ 0.05 are highlighted in yellow.

Domain	Survey	Factor	ANOSIM R	ANOSIM p
euks	G1	depth	-0.155	0.749
euks	G1	filter	0.453	0.001
euks	G1	region	0.220	0.005
euks	G2	depth	0.554	0.001
euks	G2	filter	0.088	0.002
euks	G2	region	0.346	0.001
euks	G3	depth	0.237	0.011
euks	G3	filter	0.349	0.001
euks	G3	region	0.455	0.001
proks	G1	depth	0.052	0.411
proks	G1	filter	0.240	0.002
proks	G1	region	0.188	0.012
proks	G2	depth	-0.133	0.787
proks	G2	filter	0.134	0.001
proks	G2	region	0.397	0.001
proks	G3	depth	0.099	0.202
proks	G3	filter	0.320	0.001
proks	G3	region	0.317	0.001

Table S2. EnvFit NMDS Correlations on Group Relative Percent Abundance. Results from an EnvFit analysis which was applied to major taxonomic groups of eukaryotic and prokaryotic communities across the three yearly surveys (G1 = Cruise 2016, G2 = Cruise 2017, G3 = Cruise 2019). Details on taxonomic groupings can be found in Fig S2 and 3.

EnvFit NMDS Correlations

Taxa (Continuous) Variables

Prokaryotes

Survey	Taxa	r2	p_value
G1	Actinobacteriota	0.4017	0.001
G1	Archaea	0.0100	0.840
G1	Bacteroidota	0.3729	0.002
G1	Other	0.3214	0.005
G1	Planctomycetota	0.3689	0.001
G1	Prochlorococcus	0.5345	0.001
G1	Proteobacteria	0.4819	0.002
G1	SAR406	0.4289	0.001
G1	Synechococcus	0.4469	0.001
G1	Verrucomicrobiota	0.6447	0.001
G2	Actinobacteriota	0.4473	0.001
G2	Archaea	0.3344	0.001
G2	Bacteroidota	0.4571	0.001
G2	Other	0.0675	0.121
G2	Planctomycetota	0.3859	0.001
G2	Prochlorococcus	0.5557	0.001
G2	Proteobacteria	0.2218	0.001
G2	SAR406	0.2987	0.001
G2	Synechococcus	0.4298	0.001
G2	Verrucomicrobiota	0.1903	0.003
G3	Actinobacteriota	0.0316	0.176
G3	Archaea	0.1512	0.001
G3	Bacteroidota	0.2533	0.001
G3	Other	0.3419	0.001
G3	Planctomycetota	0.1436	0.001
G3	Prochlorococcus	0.2730	0.001
G3	Proteobacteria	0.1258	0.001
G3	SAR406	0.0804	0.005
G3	Synechococcus	0.2287	0.001
G3	Verrucomicrobiota	0.1074	0.007

Eukaryotes

Survey	Taxa	r2	p_value
G1	Alveolata	0.7638	0.001
G1	Archaeplastida	0.3428	0.001
G1	Haptophyta	0.3312	0.001
G1	Opisthokonta	0.8372	0.001
G1	Other	0.2943	0.004
G1	Rhizaria	0.1834	0.019
G1	Stramenopiles	0.5232	0.001
G1	Unknown Eukaryote	0.2900	0.004
G2	Alveolata	0.2596	0.001
G2	Archaeplastida	0.7301	0.001
G2	Haptophyta	0.2257	0.001
G2	Opisthokonta	0.5000	0.001
G2	Other	0.0154	0.646
G2	Rhizaria	0.3012	0.001
G2	Stramenopiles	0.0709	0.100
G2	Unknown Eukaryote	0.2856	0.001
G3	Alveolata	0.7113	0.001
G3	Archaeplastida	0.5673	0.001
G3	Haptophyta	0.3922	0.001
G3	Opisthokonta	0.7304	0.001
G3	Other	0.1602	0.001
G3	Rhizaria	0.1035	0.003
G3	Stramenopiles	0.6061	0.001
G3	Unknown Eukaryote	0.1947	0.001

Table S3. Taxonomic Composition of Yellow and Purple WGCNA Clusters.
Taxonomy information for ASVs identified as members of the Yellow, Purple, and Teal clusters the WGCNA network. Each row is an individual ASV where cells in the ‘Cluster’ column are colored based on WGCNA cluster. Biochemical variables NCP, POC, and PON are included in the ‘ASV_ID’ column to show their cluster assignment.

Cluster	ASV_ID	Group	Phylum	Class	Order	Family	Genus	Species
Purple	ASV9576c	Stramenopiles	Bigyra	Sagenista	Sagenista_X	MAST-4	MAST-4E	MAST-4E_sp.
Purple	ASV2f69e	Archaeplastida	Chlorophyta	Mamiellophyceae	Mamiellales	Mamiellaceae	Micromonas	Micromonas_commoda_A2
Purple	ASV405f6	Archaeplastida	Chlorophyta	Mamiellophyceae	Mamiellales	Bathycoccaceae	Bathycoccus	Bathycoccus_prasinos
Purple	ASV75bed	Dinoflagellata	Dinoflagellata	Syndiniales	Dino-Group-II	Dino-Group-II-Clade-10-and-11	Dino-Group-II-Clade-10-and-11_X	Dino-Group-II-Clade-10-and-11_X_sp.
Purple	ASVfc1ec	Dinoflagellata	Dinoflagellata	Syndiniales	Dino-Group-I	Dino-Group-I-Clade-1	Dino-Group-I-Clade-1_X	Dino-Group-I-Clade-1_X_sp.
Purple	ASV7c01a	Dinoflagellata	Dinoflagellata	Syndiniales	Dino-Group-II	Dino-Group-II-Clade-10-and-11	Dino-Group-II-Clade-10-and-11_X	Dino-Group-II-Clade-10-and-11_X_sp.
Purple	ASVe8af1	Dinoflagellata	Dinoflagellata	Dinophyceae	Prorocentrales	Prorocentraceae	Prorocentrum	
Purple	ASV4963e	Dinoflagellata	Dinoflagellata	Syndiniales	Dino-Group-I	Dino-Group-I-Clade-4	Dino-Group-I-Clade-4_X	Dino-Group-I-Clade-4_X_sp.
Purple	ASV25f1c	Dinoflagellata	Dinoflagellata	Dinophyceae				
Purple	ASVab7c2	Dinoflagellata	Dinoflagellata	Dinophyceae				
Purple	ASVf6ee1	Stramenopiles	Gyrista	Pelagophyceae	Pelagomonadales	Pelagomonadaceae	Pelagomonas	Pelagomonas_calceolata
Purple	ASV9ea65	Stramenopiles	Gyrista	Dictyochophyceae	Dictyochophyceae_X	Florenciellales		
Purple	ASV4d8f7	Stramenopiles	Gyrista	Bolidophyceae	Parmales	Triparmaceae	Triparma	Triparma_pacifica
Purple	ASV0ec07	Stramenopiles	Gyrista	Pelagophyceae	Pelagomonadales	Pelagomonadaceae	Pelagomonadaceae_clade_C	Pelagomonadaceae_clade_C_sp.
Purple	ASVb472b	Stramenopiles	Gyrista	Pelagophyceae	Pelagomonadales	Pelagomonadales_clade_B	Pelagomonadales_clade_B1	Pelagomonadales_clade_B1_sp.
Purple	ASVd5d61	Stramenopiles	Gyrista	Pelagophyceae	Pelagomonadales	Pelagomonadaceae	Aureococcus	Aureococcus_anophagefferens
Purple	ASVe1e58	Stramenopiles	Gyrista	Mediophyceae	Cymatosirales	Cymatosiraceae	Brockmanniella	Brockmanniella_brockmannii
Purple	ASV482bc	Stramenopiles	Gyrista	Pelagophyceae	Pelagomonadales	Pelagomonadaceae	Pelagomonadaceae_clade_D1	Pelagomonadaceae_clade_D1_sp.
Purple	ASV06b13	Stramenopiles	Gyrista	Bacillariophyceae	Bacillariales	Bacillariaceae	Fragilariopsis	
Purple	ASVec9cb	Stramenopiles	Gyrista	Gyrista_X	Gyrista_XX	MAST-2	MAST-2D	MAST-2D_sp.
Purple	ASVc675c	Stramenopiles	Gyrista	Dictyochophyceae	Dictyochophyceae_X	Pedinellales		
Purple	ASVbb0ee	Haptophyta	Haptophyta	Prymnesiophyceae	Prymnesiophyceae_Clade_D	Prymnesiophyceae_Clade_D_X	Prymnesiophyceae_Clade_D_XX	Prymnesiophyceae_Clade_D_XX_sp.
Purple	ASV2ddf7	Haptophyta	Haptophyta	Prymnesiophyceae	Prymnesiales	Chrysochromulinaceae	Chrysochromulina	
Purple	ASV98a19	Haptophyta	Haptophyta	Prymnesiophyceae	Prymnesiales	Chrysochromulinaceae	Chrysochromulina	Chrysochromulina_sp.
Purple	ASVa9538	Haptophyta	Haptophyta	Prymnesiophyceae	Phaeocystales	Phaeocystaceae	Phaeocystis	Phaeocystis_globosa
Purple	ASV7fd9a	Haptophyta	Haptophyta	Prymnesiophyceae	Phaeocystales	Phaeocystaceae	Phaeocystis	Phaeocystis_pouchetii
Purple	ASV94519	Archaeplastida	Picozoa	Picozoa_XX	Picozoa_XXX	Picozoa_XXXX	Picozoa_XXXXX	Picozoa_XXXXX_sp.
Teal	ASVfa0b2	Dinoflagellata	Dinoflagellata	Syndiniales	Dino-Group-I	Dino-Group-I-Clade-3	Dino-Group-I-Clade-3_X	Dino-Group-I-Clade-3_X_sp.
Teal	ASV9feeb	Stramenopiles	Gyrista	Mediophyceae	Thalassiosirales	Thalassiosiraceae		
Teal	ASVd13af	Stramenopiles	Gyrista	Coscinodiscophyceae	Coscinodiscales	Hemidiscaceae	Actinocyclus	Actinocyclus_sp.
Teal	ASVb497a	Stramenopiles	Gyrista	Bacillariophyceae	Naviculales			
Yellow	ASV1c13e	Stramenopiles	Bigyra	Sagenista	Sagenista_X	MAST-7	MAST-7A	MAST-7A_sp.
Yellow	ASVa1d5e	Archaeplastida	Chlorophyta	Chloropicophyceae	Chloropicales	Chloropicaceae	Chloroparvula	Chloroparvula_pacifica
Yellow	ASV5f4ba	Synechococcus	Cyanobacteria	Cyanobacteriia	Synechococcales	Cyanobiaceae	Synechococcus_CC9902	
Yellow	ASV62139	Synechococcus	Cyanobacteria	Cyanobacteriia	Synechococcales	Cyanobiaceae	Synechococcus_CC9902	uncultured_sp
Yellow	ASV6efcd	Synechococcus	Cyanobacteria	Cyanobacteriia	Synechococcales	Cyanobiaceae	Synechococcus_CC9902	
Yellow	ASV3009d	Synechococcus	Cyanobacteria	Cyanobacteriia	Synechococcales	Cyanobiaceae	Synechococcus_CC9902	uncultured_sp
Yellow	ASV0dcda	Dinoflagellata	Dinoflagellata	Syndiniales	Dino-Group-II	Dino-Group-II-Clade-32	Dino-Group-II-Clade-32_X	Dino-Group-II-Clade-32_X_sp.
Yellow	ASV4bf35	Dinoflagellata	Dinoflagellata	Syndiniales	Dino-Group-II	Dino-Group-II-Clade-10-and-11	Dino-Group-II-Clade-10-and-11_X	Dino-Group-II-Clade-10-and-11_X_sp.
Yellow	ASV57c31	Dinoflagellata	Dinoflagellata	Syndiniales	Dino-Group-II	Dino-Group-II-Clade-7	Dino-Group-II-Clade-7_X	Dino-Group-II-Clade-7_X_sp.
Yellow	ASV85179	Dinoflagellata	Dinoflagellata	Dinophyceae				
Yellow	ASV69bae	Stramenopiles	Gyrista	Bolidophyceae	Parmales	Parmales_env_3	Parmales_env_3A	Parmales_env_3A_sp.
Yellow	ASVb10bf	Stramenopiles	Gyrista	Mediophyceae				
Yellow	ASV6f81e	Stramenopiles	Gyrista	Dictyochophyceae	Dictyochophyceae_X	Florenciellales	Pseudochattonella	Pseudochattonella_sp.
Yellow	ASV74072	Stramenopiles	Gyrista	Gyrista_X	Gyrista_XX	MAST-1	MAST-1B	MAST-1B_sp.
Yellow	ASVee3a5	Stramenopiles	Gyrista	Gyrista_X	Gyrista_XX	MAST-1	MAST-1C	MAST-1C_sp.
Yellow	ASVf6b94	Stramenopiles	Gyrista	Dictyochophyceae	Dictyochophyceae_X	Dictyochophyceae_XX	Dictyochophyceae_XXX	Dictyochophyceae_XXX_sp.
Yellow	ASVaff6d	Stramenopiles	Gyrista	Mediophyceae				
Yellow	ASV87490	Stramenopiles	Gyrista	Dictyochophyceae	Dictyochophyceae_X	Dictyochales	Dictyocha	Dictyocha_speculum
Yellow	ASV064f4	Stramenopiles	Gyrista	Pelagophyceae	Pelagomonadales	Pelagomonadales_clade_B	Pelagomonadales_clade_B1	Pelagomonadales_clade_B1_sp.
Yellow	ASVa381f	Stramenopiles	Gyrista	Dictyochophyceae	Dictyochophyceae_X	Florenciellales	Pseudochattonella	Pseudochattonella_sp.
Yellow	ASVc0b80	Stramenopiles	Gyrista	Gyrista_X	Gyrista_XX	MAST-1	MAST-1A	MAST-1A_sp.
Yellow	ASV4e146	Haptophyta	Haptophyta	Prymnesiophyceae	Calcihaptophycidae			
Yellow	ASV19672	Haptophyta	Haptophyta	Prymnesiophyceae	Prymnesiales	Chrysochromulinaceae	Chrysochromulina	Chrysochromulina_sp.
Yellow	ASV3814e	Haptophyta	Haptophyta	Haptophyta_Clade_HAP3	Haptophyta_Clade_HAP3_X	Haptophyta_Clade_HAP3_XX	Haptophyta_Clade_HAP3_XXX	Haptophyta_Clade_HAP3_XXX_sp.
Yellow	ASVf391e	Haptophyta	Haptophyta	Prymnesiophyceae	Phaeocystales	Phaeocystaceae	Phaeocystis	Phaeocystis_antarctica
Yellow	ASV9edcf	Haptophyta	Haptophyta	Prymnesiophyceae	Prymnesiales	Chrysochromulinaceae	Chrysochromulina	Chrysochromulina_sp.
Yellow	ASV23dac	Archaeplastida	Picozoa	Picozoa_XX	Picozoa_XXX	Picozoa_XXXX	Picozoa_XXXXX	Picozoa_XXXXX_sp.
Yellow	ASV077d6	Archaeplastida	Prasinodermophyta	Prasinodermophyceae	Prasinodermales	Prasinodermaceae	Prasinoderma	
Yellow	NCP							
Yellow	POC							
Yellow	PON							

Table S4. Top 5 Out-degree ASVs for Prokaryotes (16S) and Eukaryotes (18S)

ASVs with the highest connectivity are highlighted in blue (16S prokaryotes) and green (18S eukaryotes). Outdegree values represent the number of first-degree connections (network edges) for each ASV in the overall SpiecEasi co-occurrence network. ASVs with high outdegree have the most connections in the network. Taxonomic classifications are provided.

Top 5 OutDegree ASVs

Cytoscape Network Analysis Tool

Group	Domain	ASV ID	Outdegree	Phylum	Class	Order	Family	Genus	Species
Proteobacteria	16S	ASV7d83e	60	Proteobacteria	Alphaproteobacteria	SAR11_clade	Clade_II	Clade_II	NA
Bacteroidota	16S	ASV2c871	59	Bacteroidota	Bacteroidia	Flavobacteriales	Flavobacteriaceae	NS5_marine_group	NA
Proteobacteria	16S	ASVaac3f	56	Proteobacteria	Alphaproteobacteria	SAR11_clade	Clade_II	Clade_II	NA
Actinobacteria	16S	ASV74a26	55	Actinobacteriota	Actinobacteria	Corynebacteriales	Mycobacteriaceae	Mycobacterium	NA
Proteobacteria	16S	ASV2c674	54	Proteobacteria	Alphaproteobacteria	SAR11_clade	Clade_I	Clade_Ia	NA
Haptophyta	18S	ASV41683	47	Haptophyta	NA	NA	NA	NA	NA
Archaeplastida	18S	ASVa1d5e	44	Chlorophyta	Chloropicophyceae	Chloropicales	Chloropicaceae	Chloroparvula	Chloroparvula_pacifica
Dinoflagellata	18S	ASV00a36	42	Dinoflagellata	Syndiniales	Dino-Group-I	Dino-Group-I-Clade-1	Dino-Group-I-Clade-1_X	Dino-Group-I-Clade-1_X_sp.
Stramenopiles	18S	ASVb3907	41	Gyrista	Dictyochophyceae	Dictyochophyceae_X	Dictyochales	Dictyocha	Dictyocha_globosa
Archaeplastida	18S	ASV94519	41	Picozoa	Picozoa_XX	Picozoa_XXX	Picozoa_XXXX	Picozoa_XXXXX	Picozoa_XXXXX_sp.

Table S5. Yellow and Purple Candidate Connections from Spiec-Easi Network. Top positive-weight connections between yellow and purple WGCNA candidate ASVs and their first-degree neighbors. ASVs are color-coded by their WGCNA cluster assignments. Taxonomic classifications (Family | Genus | Species) are provided. Gray-colored cells indicate ASVs that were not assigned to a WGCNA module (i.e. not present in the WGCNA network results).

Candidate Cluster	Candidate ASV ID	Candidate Group	Candidate Taxonomy (Family : Species)	Weight	Associate Cluster	Associate ASV ID	Associate Domain	Associate Group	Associate Taxonomy (Family : Species)
Purple	asv06b13	Stramenopiles	Bacillariaceae | Fragilariopsis | NA	0.10	Purple	asv7fd9a	Euk	Haptophyta	Phaeocystaceae | Phaeocystis | Phaeocystis_pouchetii
Purple	asv405f6	Archaeplastida	Bathycoccaceae | Bathycoccus | Bathycoccus_prasinos	0.16	Purple	asvbb0ee	Euk	Haptophyta	Prymnesiophyceae_Clade_D_X | Prymnesiophyceae_Clade_D_XX | Prymnesiophyceae_Clade_D_XX_sp.
Purple	asv98a19	Haptophyta	Chrysochromulinaceae | Chrysochromulina | Chrysochromulina_sp.	0.19	Gray	asv705d3	Euk	Dinoflagellata	NA | NA | NA
Purple	asv2ddf7	Haptophyta	Chrysochromulinaceae | Chrysochromulina | NA	0.19	Unassigned	asv02ae7	Euk	Dinoflagellata	Dino-Group-II-Clade-10-and-11 | Dino-Group-II-Clade-10-and-11_X | Dino-Group-II-Clade-10-and-11_X_sp.
Purple	asve1e58	Stramenopiles	Cymatosiraceae | Brockmanniella | Brockmanniella_brockmannii	0.12	Yellow	asv87490	Euk	Stramenopiles	Dictyochales | Dictyocha | Dictyocha_speculum
Purple	asvfc1ec	Dinoflagellata	Dino-Group-I-Clade-1 | Dino-Group-I-Clade-1_X | Dino-Group-I-Clade-1_X_sp.	0.09	Purple	asv4963e	Euk	Dinoflagellata	Dino-Group-I-Clade-4 | Dino-Group-I-Clade-4_X | Dino-Group-I-Clade-4_X_sp.
Purple	asv4963e	Dinoflagellata	Dino-Group-I-Clade-4 | Dino-Group-I-Clade-4_X | Dino-Group-I-Clade-4_X_sp.	0.28	Pink	asv3b1aa	Euk	Archaeplastida	Chloropicaceae | Chloroparvula | Chloroparvula_B3_sp.
Purple	asv75bed	Dinoflagellata	Dino-Group-II-Clade-10-and-11 | Dino-Group-II-Clade-10-and-11_X | Dino-Group-II-Clade-10-and-11_X_sp.	0.01	Unassigned	asv3b6d6	Euk	Dinoflagellata	Dino-Group-I-Clade-4 | Dino-Group-I-Clade-4_X | Dino-Group-I-Clade-4_X_sp.
Purple	asv7c01a	Dinoflagellata	Dino-Group-II-Clade-10-and-11 | Dino-Group-II-Clade-10-and-11_X | Dino-Group-II-Clade-10-and-11_X_sp.	0.06	Unassigned	asv097e8	Euk	Dinoflagellata	NA | NA | NA
Purple	asv9ea65	Stramenopiles	Florenciellales | NA | NA	0.19	Purple	asv25f1c	Euk	Dinoflagellata	NA | NA | NA
Purple	asvec9cb	Stramenopiles	MAST-2 | MAST-2D | MAST-2D_sp.	0.07	Yellow	asvee3a5	Euk	Stramenopiles	MAST-1 | MAST-1C | MAST-1C_sp.
Purple	asv9576c	Stramenopiles	MAST-4 | MAST-4E | MAST-4E_sp.	0.13	Gray	asv68b9e	Euk	Haptophyta	Chrysochromulinaceae | Chrysochromulina | Chrysochromulina_sp.
Purple	asv2f69e	Archaeplastida	Mamiellaceae | Micromonas | Micromonas_commoda_A2	0.28	Purple	asv7fd9a	Euk	Haptophyta	Phaeocystaceae | Phaeocystis | Phaeocystis_pouchetii
Purple	asv25f1c	Dinoflagellata	NA | NA | NA	0.07	Yellow	asv69bae	Euk	Stramenopiles	Parmales_env_3 | Parmales_env_3A | Parmales_env_3A_sp.
Purple	asvc675c	Stramenopiles	Pedinellales | NA | NA	0.07	Yellow	asv87490	Euk	Stramenopiles	Dictyochales | Dictyocha | Dictyocha_speculum
Purple	asvd5d61	Stramenopiles	Pelagomonadaceae | Aureococcus | Aureococcus_anophagefferens	0.08	Purple	asve1e58	Euk	Stramenopiles	Cymatosiraceae | Brockmanniella | Brockmanniella_brockmannii
Purple	asv0ec07	Stramenopiles	Pelagomonadaceae | Pelagomonadaceae_clade_C | Pelagomonadaceae_clade_C_sp.	0.14	Purple	asvb472b	Euk	Stramenopiles	Pelagomonadales_clade_B | Pelagomonadales_clade_B1 | Pelagomonadales_clade_B1_sp.
Purple	asv482bc	Stramenopiles	Pelagomonadaceae | Pelagomonadaceae_clade_D1 | Pelagomonadaceae_clade_D1_sp.	0.01	Unassigned	asv1b4cd	Euk	Archaeplastida	Halosphaeraceae | Halosphaera | Halosphaera_sp.
Purple	asvf6ee1	Stramenopiles	Pelagomonadaceae | Pelagomonas | Pelagomonas_calceolata	0.45	Purple	asv405f6	Euk	Archaeplastida	Bathycoccaceae | Bathycoccus | Bathycoccus_prasinos
Purple	asvb472b	Stramenopiles	Pelagomonadales_clade_B | Pelagomonadales_clade_B1 | Pelagomonadales_clade_B1_sp.	0.12	Purple	asv7fd9a	Euk	Haptophyta	Phaeocystaceae | Phaeocystis | Phaeocystis_pouchetii
Purple	asva9538	Haptophyta	Phaeocystaceae | Phaeocystis | Phaeocystis_globosa	0.05	Unassigned	asvb7db9	Euk	Haptophyta	Chrysochromulinaceae | Chrysochromulina | NA
Purple	asv7fd9a	Haptophyta	Phaeocystaceae | Phaeocystis | Phaeocystis_pouchetii	0.27	Yellow	asvf391e	Euk	Haptophyta	Phaeocystaceae | Phaeocystis | Phaeocystis_antarctica
Purple	asv94519	Archaeplastida	Picozoa_XXXX | Picozoa_XXXXX | Picozoa_XXXXX_sp.	0.06	Yellow	asv4e146	Euk	Haptophyta	NA | NA | NA
Purple	asve8af1	Dinoflagellata	Prorocentraceae | Prorocentrum | NA	0.13	Sky	asv3c041	Euk	Haptophyta	Phaeocystaceae | Phaeocystis | NA
Purple	asvbb0ee	Haptophyta	Prymnesiophyceae_Clade_D_X | Prymnesiophyceae_Clade_D_XX | Prymnesiophyceae_Clade_D_XX_sp.	0.20	Purple	asv2ddf7	Euk	Haptophyta	Chrysochromulinaceae | Chrysochromulina | NA
Purple	asv4d8f7	Stramenopiles	Triparmaceae | Triparma | Triparma_pacifica	0.06	Purple	asv94519	Euk	Archaeplastida	Picozoa_XXXX | Picozoa_XXXXX | Picozoa_XXXXX_sp.
Sky	asv180a1	Haptophyta	Braarudosphaeraceae | Braarudosphaeraceae_X | Braarudosphaeraceae_X_sp.	0.18	Sky	asvc701f	Euk	Haptophyta	Braarudosphaeraceae | Braarudosphaera | Braarudosphaera_bigelowii
Sky	asv180a1	Haptophyta	Braarudosphaeraceae | Braarudosphaeraceae_X | Braarudosphaeraceae_X_sp.	0.22	Unassigned	asv4d1ff	Prok	UCYN_A	Microcystaceae | Atelocyanobacterium_(UCYN-A) | Candidatus_Atelocyanobacterium
Yellow	asva1d5e	Archaeplastida	Chloropicaceae | Chloroparvula | Chloroparvula_pacifica	0.37	Pink	asvb10a4	Euk	Dinoflagellata	Dino-Group-I-Clade-1 | Dino-Group-I-Clade-1_X | Dino-Group-I-Clade-1_X_sp.
Yellow	asva1d5e	Archaeplastida	Chloropicaceae | Chloroparvula | Chloroparvula_pacifica	0.10	Unassigned	asv0f593	Prok	Bacteroidota	Flavobacteriaceae | NS2b_marine_group | NA
Yellow	asv19672	Haptophyta	Chrysochromulinaceae | Chrysochromulina | Chrysochromulina_sp.	0.05	Unassigned	asv1ce61	Euk	Dinoflagellata	Dino-Group-II-Clade-6 | Dino-Group-II-Clade-6_X | Dino-Group-II-Clade-6_X_sp.
Yellow	asv9edcf	Haptophyta	Chrysochromulinaceae | Chrysochromulina | Chrysochromulina_sp.	0.18	Yellow	asvc0b80	Euk	Stramenopiles	MAST-1 | MAST-1A | MAST-1A_sp.
Yellow	asv5f4ba	Synechococcus	Cyanobiaceae | Synechococcus_CC9902 | NA	0.15	Teal	asv9feeb	Euk	Stramenopiles	Thalassiosiraceae | NA | NA
Yellow	asv5f4ba	Synechococcus	Cyanobiaceae | Synechococcus_CC9902 | NA	0.12	Yellow	asv6efcd	Prok	Synechococcus	Cyanobiaceae | Synechococcus_CC9902 | NA
Yellow	asv6efcd	Synechococcus	Cyanobiaceae | Synechococcus_CC9902 | NA	0.05	Purple	asve1e58	Euk	Stramenopiles	Cymatosiraceae | Brockmanniella | Brockmanniella_brockmannii
Yellow	asv3009d	Synechococcus	Cyanobiaceae | Synechococcus_CC9902 | uncultured_sp	0.22	Unassigned	asv357e8	Euk	Stramenopiles	Chrysophyceae_Clade-EC2H_X | Chrysophyceae_Clade-EC2H_XX | Chrysophyceae_Clade-EC2H_XX_sp.
Yellow	asv3009d	Synechococcus	Cyanobiaceae | Synechococcus_CC9902 | uncultured_sp	0.14	Unassigned	asv3b154	Prok	Alphaproteobacteria	Clade_I | NA | NA
Yellow	asv62139	Synechococcus	Cyanobiaceae | Synechococcus_CC9902 | uncultured_sp	0.14	Yellow	asv69bae	Euk	Stramenopiles	Parmales_env_3 | Parmales_env_3A | Parmales_env_3A_sp.
Yellow	asv62139	Synechococcus	Cyanobiaceae | Synechococcus_CC9902 | uncultured_sp	0.07	Unassigned	asv2d102	Prok	Cyanobacteria	Cyanobiaceae | NA | NA
Yellow	asv87490	Stramenopiles	Dictyochales | Dictyocha | Dictyocha_speculum	0.07	Unassigned	asv7ac0a	Euk	Dinoflagellata	NA | NA | NA
Yellow	asvf6b94	Stramenopiles	Dictyochophyceae_XX | Dictyochophyceae_XXX | Dictyochophyceae_XXX_sp.	0.14	Yellow	asv57c31	Euk	Dinoflagellata	Dino-Group-II-Clade-7 | Dino-Group-II-Clade-7_X | Dino-Group-II-Clade-7_X_sp.
Yellow	asv4bf35	Dinoflagellata	Dino-Group-II-Clade-10-and-11 | Dino-Group-II-Clade-10-and-11_X | Dino-Group-II-Clade-10-and-11_X_sp.	0.13	Yellow	asv69bae	Euk	Stramenopiles	Parmales_env_3 | Parmales_env_3A | Parmales_env_3A_sp.
Yellow	asv0dcda	Dinoflagellata	Dino-Group-II-Clade-32 | Dino-Group-II-Clade-32_X | Dino-Group-II-Clade-32_X_sp.	0.13	Purple	asv2ddf7	Euk	Haptophyta	Chrysochromulinaceae | Chrysochromulina | NA
Yellow	asv57c31	Dinoflagellata	Dino-Group-II-Clade-7 | Dino-Group-II-Clade-7_X | Dino-Group-II-Clade-7_X_sp.	0.20	Yellow	asva381f	Euk	Stramenopiles	Florenciellales | Pseudochattonella | Pseudochattonella_sp.
Yellow	asv6f81e	Stramenopiles	Florenciellales | Pseudochattonella | Pseudochattonella_sp.	0.06	Yellow	asv3814e	Euk	Haptophyta	Haptophyta_Clade_HAP3_XX | Haptophyta_Clade_HAP3_XXX | Haptophyta_Clade_HAP3_XXX_sp.
Yellow	asva381f	Stramenopiles	Florenciellales | Pseudochattonella | Pseudochattonella_sp.	0.08	Unassigned	asv73f97	Euk	Stramenopiles	Thalassiosiraceae | Thalassiosira | NA
Yellow	asv3814e	Haptophyta	Haptophyta_Clade_HAP3_XX | Haptophyta_Clade_HAP3_XXX | Haptophyta_Clade_HAP3_XXX_sp.	0.02	Yellow	asva381f	Euk	Stramenopiles	Florenciellales | Pseudochattonella | Pseudochattonella_sp.
Yellow	asv74072	Stramenopiles	MAST-1 | MAST-1B | MAST-1B_sp.	0.40	Yellow	asvee3a5	Euk	Stramenopiles	MAST-1 | MAST-1C | MAST-1C_sp.
Yellow	asvee3a5	Stramenopiles	MAST-1 | MAST-1C | MAST-1C_sp.	0.11	Yellow	asvf6b94	Euk	Stramenopiles	Dictyochophyceae_XX | Dictyochophyceae_XXX | Dictyochophyceae_XXX_sp.
Yellow	asv1c13e	Stramenopiles	MAST-7 | MAST-7A | MAST-7A_sp.	0.05	Yellow	asvc0b80	Euk	Stramenopiles	MAST-1 | MAST-1A | MAST-1A_sp.
Yellow	asv4e146	Haptophyta	NA | NA | NA	0.13	Unassigned	asv357e8	Euk	Stramenopiles	Chrysophyceae_Clade-EC2H_X | Chrysophyceae_Clade-EC2H_XX | Chrysophyceae_Clade-EC2H_XX_sp.
Yellow	asv85179	Dinoflagellata	NA | NA | NA	0.05	Unassigned	asv677ab	Euk	Dinoflagellata	Dino-Group-II-Clade-1 | Dino-Group-II-Clade-1_X | Dino-Group-II-Clade-1_X_sp.
Yellow	asvaff6d	Stramenopiles	NA | NA | NA	0.29	Yellow	asv9edcf	Euk	Haptophyta	Chrysochromulinaceae | Chrysochromulina | Chrysochromulina_sp.
Yellow	asvb10bf	Stramenopiles	NA | NA | NA	0.16	Unassigned	asvb7db9	Euk	Haptophyta	Chrysochromulinaceae | Chrysochromulina | NA
Yellow	asv69bae	Stramenopiles	Parmales_env_3 | Parmales_env_3A | Parmales_env_3A_sp.	0.10	Yellow	asvc0b80	Euk	Stramenopiles	MAST-1 | MAST-1A | MAST-1A_sp.
Yellow	asvf391e	Haptophyta	Phaeocystaceae | Phaeocystis | Phaeocystis_antarctica	0.04	Yellow	asv9edcf	Euk	Haptophyta	Chrysochromulinaceae | Chrysochromulina | Chrysochromulina_sp.
Yellow	asv23dac	Archaeplastida	Picozoa_XXXX | Picozoa_XXXXX | Picozoa_XXXXX_sp.	0.18	Unassigned	asvcf9da	Euk	Dinoflagellata	Dino-Group-III_X | Dino-Group-III_XX | Dino-Group-III_XX_sp.
Yellow	asv23dac	Archaeplastida	Picozoa_XXXX | Picozoa_XXXXX | Picozoa_XXXXX_sp.	0.09	Unassigned	asv8cff7	Prok	Gammaproteobacteria	Halieaceae | OM60(NOR5)_clade | NA
Yellow	asv077d6	Archaeplastida	Prasinodermaceae | Prasinoderma | NA	0.17	Purple	asv0ec07	Euk	Stramenopiles	Pelagomonadaceae | Pelagomonadaceae_clade_C | Pelagomonadaceae_clade_C_sp.
Yellow	asv077d6	Archaeplastida	Prasinodermaceae | Prasinoderma | NA	0.09	Unassigned	asv39fa7	Prok	Bacteroidota	Saprospiraceae | Aureispira | NA

Table S6. Rarefaction Thresholds for 18S and 16S Amplicon Sequencing Across the Yearly Surveys. The table shows rarefaction thresholds applied to eukaryotic 18S and prokaryotic 16S ASV datasets for three yearly surveys collected during 2016–2019 survey years. Thresholds were selected based on sequencing depth to ensure comparability across samples. Cruise 2016 (G1) is represented in green, Cruise 2017 (G2) in orange, and Cruise 2019 (G3) in purple.

Cruise	Prokaryotes_16S	Eukaryotes_18S
2016_Gradients1	90,000	35,000
2017_Gradients2	20,000	10,000
2019_Gradients3	50,000	70,000

SUPPLEMENTAL DATASET FILES

S1: Sample Site Meta (Replicates)

S2: Spiec-Easi Associations

Available on https://github.com/rkeyMicrobe/picoGrads2025

SUPPLEMENTAL MATERIALS AND METHODS

Amplicon Processing

ASVs were processed using QIIME2 v2022.8 (2). Quality control on paired-end reads were conducted with FastQC v0.11.9 (3), followed by FIGARO (v1.0.0) to determine optimal trimming parameters for 16S reads. DADA2 v1.14.1 (4), implemented within QIIME2, was used to merge paired-end reads using FIGARO parameters and a maximum error rate of 0–2. Of note, the V4-V5 reads from the 18S rDNA region could not be merged, so V4 reads that didn't exceed a maximum error rate over 2 were used. Taxonomic classification was performed using the Qiime2 plug-in, RESCRIPt (v2022.8.0). For 16S, a naive Bayes classifier trained on the SILVA database v138.1 (5) targeting the V4 region (~309 bp) was used. For 18S, classification targeted the V4–V5 region (~635 bp) using a Mothur-formatted PR2 database v5.1 (6, 7). For each cruise and rRNA type, three outputs were generated: ASV count tables, taxonomic assignments, and ASV sequences, which were imported into R for downstream analysis. In R-studio (v4.4.0, Puppy Cup), rarefaction curves were made to select appropriate sequencing depth thresholds (Table S6). Samples were rarefied with `rarefy_even_depth()` using phyloseq v1.44.0 (8).

Community Analysis

Microbial community analyses were conducted using the vegan v2.6-4 (9), phyloseq v1.44.0 (8), and tidyverse v2.0.0 (10) packages. We first calculated relative abundances for all ASVs by dividing ASV count observations by pre-determined rarefy thresholds (Table S6). Non-metric Multidimensional Scaling (NMDS) was performed using *vegan* v2.6-4. ANOSIM was used to test for significant differences in community composition between groups defined by filter size, depth, and region across the three survey years. Post-hoc fitting of continuous variables (e.g. taxonomic groups) was performed on the NMDS ordination using the 'envfit' function to quantify how filter size, latitude, and phyla-level taxonomy explained compositional variance (R^2) of samples.

ASVs representing the collective community were categorized as either persistent (present in all three cruises) or ephemeral (present in only one or two cruises). To quantify how distinct each category was from the total community (persistent + ephemeral), Bray-Curtis dissimilarities were calculated between ephemeral vs. collective and persistent vs. collective groups. For each sample, ASV-level relative percent abundances were summed by phytoplankton group, and a pseudocount of 0.0001 was added to all values to avoid zeros. Dissimilarity was computed using vegdist(method = "bray").

Physio- and Biogeochemical Data Integrations

Physiochemical measurements (ie. seawater surface temperature and seawater surface salinity) and biogeochemical measurements particulate organic carbon (POC), particulate organic nitrogen (PON), net community production (NCP) were obtained for the Simons Collaborative Marine Atlas Project (CMAP; https://simonscmap.com/ ; (11)) and correspond to data from Juranek *et al.* (1). Data were downloaded from the following links.

G1 NCP: https://simonscmap.com/catalog/datasets/KOK1606_Gradients1_Surface_O2Ar_NCP
G2 NCP: https://simonscmap.com/catalog/datasets/MGL1704_Gradients2_Surface_O2Ar_NCP
G3 NCP: https://simonscmap.com/catalog/datasets/KM1906_Gradients3_Surface_O2Ar_NCP

G1 POC/N: https://simonscmap.com/catalog/datasets/Gradients1_KOK1606_PPPCPN_UW
G2 POC/N: https://simonscmap.com/catalog/datasets/Gradients2_MGL1704_PPPCPN_UW
G3 POC/N: https://simonscmap.com/catalog/datasets/Gradients3_KM1906_PCPN_UW

Amplicon libraries were paired with corresponding POC, PON, or NCP data using a collection time cutoff of 12 hours and location cutoff of 0.5 degrees of latitude. Only those amplicon libraries that could be linked to at least one type of data were used for subsequent analyses. Using this criterion, all 48 samples from 2016 had matched NCP data, while 38 had matched POC and PON data. In 2017, 56 of 72 samples had matched NCP, and 60 had matched POC and PON data. In 2019, 168 of 192 samples had matched NCP, while all had matched POC and PON data. Among paired samples, 94% of NCP and 70% of POC/PON matches fell within 0–4 hours, while 95% of NCP and 68% of POC/PON fell within 0.2° latitude (Figure S5).

In addition, continuous SeaFlow measurements from the ship underway intake (5–7 m depth) during the G3 cruise that estimated *Prochlorococcus* and *Synechococcus* biomass Ribalet et al. (2019) (12) were compared to 16S ASV relative abundances (Figure S6).

Multi-level Mixed Modeling

To determine which phytoplankton groups best explained POC, PON, and NCP levels, we performed multivariate linear mixed modeling (MLMM) using the mmer(..., getPEV = TRUE, rcov = ~units) command within the sommer package v4.3.3 (13). ASV relative percent abundance was used to construct dissimilarity-based G-matrices for each phytoplankton group: persistent members housed within *Prochlorococcus*, *Synechococcus*, Archaeplastida, Haptophyta, Dinoflagellata within the Alveolata, and Stramenopiles belonging to Dictyochophyceae, diatoms (Coscinodiscophyceae, Bacillariophyceae, and Mediophyceae), Chrysophyceae, Pelagophyceae, Bolidophyceae, and Pinguiophyceae classes. To do this, we first added a pseudo-count of 1e-6 to all count observations. A center-log ratio (clr) was applied to each dataset, after which all data were merged to one master dataframe. Persistent phytoplankton taxa were retained while other ASVs were removed. For each phytoplankton group, a sample-by-ASV matrix was constructed from the master dataframe to calculate pairwise Euclidean distances which yielded a dissimilarity matrix per group. To avoid computational instability from zero distances, a small constant (0.001) was added to the diagonal - a regularization step commonly used to stabilize covariance matrix estimation in mixed modeling (14, 15).

We used the following equation to model the relationship between microbial community structure and biogeochemical variability across the North Pacific transect.

(a) $Y = \beta_0 + \beta_1 X_1 + \beta_2 X_2 + \beta_3 X_3 + Z_1\gamma_1 + Z_2\gamma_2 + Z_3\gamma_3 + Z_4\gamma_4 + Z_5\gamma_5 + Z_6\gamma_6 + \varepsilon$

(b) Feature ∼ time + filter + depth +
(1 | Archaeplastida) + (1 | Haptophyta) + (1 | Dinoflagellata) +
(1 | Stramenopiles) + (1 | Prochlorococcus) + (1 | Synechococcus)

where the Y represents the response variable (either POC, PON, or NCP), modeled individually and Box-Cox transformed using the bc_transform function from the bestNormalize package. X_1, X_2, X_3 correspond to fixed effects: time (Cruise × Month), filter (size fraction), and depth (binned into 0–15 m, 45–75 m, and 90–125 m ranges). $Z_n\gamma_n$ are the random effects representing dissimilarity-based G-matrices for each major phytoplankton group: Archaeplastida, Haptophyta, Dinoflagellata, Stramenopiles, *Prochlorococcus*, and *Synechococcus*. ε is the residual error term. Fixed effects were selected based on AIC and BIC minimization after testing multiple combinations. Model convergence

was assessed using the summary() function in the sommer package, which reports convergence status and AIC/BIC values. For each model, we further examined residual distributions to confirm model assumptions. Residuals and fitted values were extracted and visualized using custom plotting functions that generated residuals vs. fitted value plots (to assess homoscedasticity), histograms with Shapiro-Wilk tests (to evaluate normality), and quantile-quantile (QQ) plots of residuals to assess distributional fit and model adequacy.

Network Construction and Analysis

Weighted gene co-expression network analysis (WGCNA v1.72-5 (16) was applied to persistent ASVs from phytoplankton-containing taxa and Box-Cox–transformed NCP , POC, and PON values to identify ASV modules correlated with these biochemical variables. ASV counts were processed identically to MLMM (pseudocount addition + CLR transformation + persistence filter) to ensure consistency across approaches. To reduce sparsity, we removed rare ASVs by applying a prevalence threshold of ≥3 reads in ≥10% of samples within each cruise year. A soft-thresholding power of 6 was selected using pickSoftThreshold (scale-free topology fit > 0.8; mean connectivity > 0). Networks were constructed with blockwiseModules (TOMType = "signed", deepSplit = 4, minModuleSize = 10, mergeCutHeight = 0.25) using a biweight midcorrelation (bicor) matrix to reduce outlier effects.

ASVs were hierarchically clustered based on relative percent abundance to define module memberships, and eigengenes were computed with moduleEigengenes(). Module-trait correlations with NCP, POC, and PON were assessed using Pearson correlation, where correlations reflect statistical associations but not causality. Module composition was summarized by tallying ASVs per phytoplankton group, including species-level counts for Archaeplastida. Network structure was exported to Cytoscape v3.10.2 (17) for visualization and analysis using the ‘Network Analyzer’ tool. Additional soft-thresholding tests (powers 1–8) were run to evaluate the consistency of Archaeplastida presence in modules positively associated with NCP, POC, and PON across different network topologies.

Sparse Inverse Covariance Estimation for Ecological Association Inference (Spiec-Easi) v1.1.0 (18) was used to infer direct associations between specific persistent phytoplankton and prokaryotic ASVs (Dataset S2). Prokaryotic and persistent Eukaryotic ASVs underwent the same preprocessing (pseudocount + CLR + persistence filter) as MLMM and WGCNA before being combined. SpiecEasi networks were then generated using the Meinshausen-Bühlmann (MB) method with lambda.min.ratio = 0.01 and nlambda = 20. The Stability Approach to Regularization Selection (StARS) with 50 subsampling iterations was used to select optimal sparsity parameters. Edges representing strong positive or negative associations (> +0.1 or < –0.1) were retained for visualization. To assess spatial relevance, we examined the latitude-specific distributions of Archaeplastida and other ASVs (identified in MLMM and WGCNA) and their first neighbors in the SpiecEasi network.

Supplemental References

1. Juranek LW, White AE, Dugenne M, Henderikx Freitas F, Dutkiewicz S, Ribalet F, Ferrón S, Armbrust EV, Karl DM. 2020. The Importance of the Phytoplankton “Middle Class” to Ocean Net Community Production. Glob Biogeochem Cycles 34:e2020GB006702.

2. Bolyen E, Rideout JR, Dillon MR, Bokulich NA, Abnet CC, Al-Ghalith GA, Alexander H, Alm EJ, Arumugam M, Asnicar F, Bai Y, Bisanz JE, Bittinger K, Brejnrod A, Brislawn CJ, Brown CT, Callahan BJ, Caraballo-Rodríguez AM, Chase J, Cope EK, Da Silva R, Diener C, Dorrestein PC, Douglas GM, Durall DM, Duvallet C, Edwardson CF, Ernst M, Estaki M, Fouquier J, Gauglitz JM, Gibbons SM, Gibson DL, Gonzalez A, Gorlick K, Guo J, Hillmann B, Holmes S, Holste H, Huttenhower C, Huttley GA, Janssen S, Jarmusch AK, Jiang L, Kaehler BD, Kang KB, Keefe CR, Keim P, Kelley ST, Knights D, Koester I, Kosciolek T, Kreps J, Langille MGI, Lee J, Ley R, Liu Y-X, Loftfield E, Lozupone C, Maher M, Marotz C, Martin BD, McDonald D, McIver LJ, Melnik AV, Metcalf JL, Morgan SC, Morton JT, Naimey AT, Navas-Molina JA, Nothias LF, Orchanian SB, Pearson T, Peoples SL, Petras D, Preuss ML, Pruesse E, Rasmussen LB, Rivers A, Robeson MS, Rosenthal P, Segata N, Shaffer M, Shiffer A, Sinha R, Song SJ, Spear JR, Swafford AD, Thompson LR, Torres PJ, Trinh P, Tripathi A, Turnbaugh PJ, Ul-Hasan S, van der Hooft JJJ, Vargas F, Vázquez-Baeza Y, Vogtmann E, von Hippel M, Walters W, Wan Y, Wang M, Warren J, Weber KC, Williamson CHD, Willis AD, Xu ZZ, Zaneveld JR, Zhang Y, Zhu Q, Knight R, Caporaso JG. 2019. Author Correction: Reproducible, interactive, scalable and extensible microbiome data science using QIIME 2. Nat Biotechnol 37:1091–1091.

3. Andrews S. Babraham Bioinformatics - FastQC A Quality Control tool for High Throughput Sequence Data. https://www.bioinformatics.babraham.ac.uk/projects/fastqc/. Retrieved 28 March 2025.

4. Callahan BJ, McMurdie PJ, Rosen MJ, Han AW, Johnson AJA, Holmes SP. 2016. DADA2: High resolution sample inference from Illumina amplicon data. Nat Methods 13:581–583.

5. Quast C, Pruesse E, Yilmaz P, Gerken J, Schweer T, Yarza P, Peplies J, Glöckner FO. 2013. The SILVA ribosomal RNA gene database project: improved data processing and web-based tools. Nucleic Acids Res 41:D590–D596.

6. Schloss PD, Westcott SL, Ryabin T, Hall JR, Hartmann M, Hollister EB, Lesniewski RA, Oakley BB, Parks DH, Robinson CJ, Sahl JW, Stres B, Thallinger GG, Van Horn DJ, Weber CF. 2009. Introducing mothur: Open-Source, Platform-Independent, Community-Supported Software for Describing and Comparing Microbial Communities. Appl Environ Microbiol 75:7537–7541.

7. Guillou L, Bachar D, Audic S, Bass D, Berney C, Bittner L, Boutte C, Burgaud G, De Vargas C, Decelle J, Del Campo J, Dolan JR, Dunthorn M, Edvardsen B, Holzmann M, Kooistra WHCF, Lara E, Le Bescot N, Logares R, Mahé F, Massana R, Montresor M, Morard R, Not F, Pawlowski J, Probert I, Sauvadet A-L, Siano R, Stoeck T, Vaulot D, Zimmermann P, Christen R. 2012. The Protist Ribosomal Reference database (PR2): a catalog of unicellular eukaryote Small Sub-Unit rRNA sequences with curated taxonomy. Nucleic Acids Res 41:D597–D604.

8. McMurdie PJ, Holmes S. 2013. phyloseq: An R Package for Reproducible Interactive Analysis and Graphics of Microbiome Census Data. PLoS ONE 8:e61217.

9. Oksanen J, Simpson GL, Blanchet FG, Kindt R, Legendre P, Minchin PR, O'Hara RB, Solymos P, Stevens MHH, Szoecs E, Wagner H, Barbour M, Bedward M, Bolker B, Borcard D, Carvalho G, Chirico M, Caceres MD, Durand S, Evangelista HBA, FitzJohn R, Friendly M, Furneaux B, Hannigan G, Hill MO, Lahti L, McGlinn D, Ouellette M-H, Cunha ER, Smith T, Stier A, Braak CJFT, Weedon J, Borman T. 2025. vegan: Community Ecology Package (2.6-10).

10. Wickham H, Averick M, Bryan J, Chang W, McGowan LD, François R, Grolemund G, Hayes A, Henry L, Hester J, Kuhn M, Pedersen TL, Miller E, Bache SM, Müller K, Ooms J, Robinson D, Seidel DP, Spinu V, Takahashi K, Vaughan D, Wilke C, Woo K, Yutani H. 2019. Welcome to the Tidyverse. J Open Source Softw 4:1686.

11. Ashkezari MD, Hagen NR, Denholtz M, Neang A, Burns TC, Morales RL, Lee CP, Hill CN, Armbrust EV. 2021. Simons Collaborative Marine Atlas Project (Simons CMAP): An open-source portal to share, visualize, and analyze ocean data. Limnol Oceanogr Methods 19:488–496.

12. Ribalet, F., Berthiaume, C., Hynes, A., Swalwell, J., Carlson, M., Clayton, S., … Armbrust, E. V. (2019). *SeaFlow data v1, high-resolution abundance, size and biomass of small phytoplankton in the North Pacific*. Scientific Data, 6, Article 277.

13. Covarrubias-Pazaran G. 2016. Genome-Assisted Prediction of Quantitative Traits Using the R Package sommer. PLOS ONE 11:e0156744.

14. Speed D, Balding DJ. 2014. MultiBLUP: improved SNP-based prediction for complex traits. Genome Res 24:1550–1557.

15. Bickel PJ, Levina E. 2008. Regularized estimation of large covariance matrices. Ann Stat 36:199–227.

16. Langfelder P, Horvath S. 2008. WGCNA: an R package for weighted correlation network analysis. BMC Bioinformatics 9:559.

17. Shannon P, Markiel A, Ozier O, Baliga NS, Wang JT, Ramage D, Amin N, Schwikowski B, Ideker T. 2003. Cytoscape: A Software Environment for Integrated Models of Biomolecular Interaction Networks. Genome Res 13:2498–2504.

18. Kurtz ZD, Müller CL, Miraldi ER, Littman DR, Blaser MJ, Bonneau RA. 2015. Sparse and Compositionally Robust Inference of Microbial Ecological Networks. PLOS Comput Biol 11:e1004226.
